# Supplementary material for: Caldesmon controls stress fiber force-balance through dynamic cross-linking of myosin II and actin-tropomyosin filaments
Source: Nat Commun. 2022 Oct 13;13:6032. doi: 10.1038/s41467-022-33688-w (PMC9561149; doi:10.1038/s41467-022-33688-w)
Supplement: Supplementary file 1 — Supplementary information [file 41467_2022_33688_MOESM1_ESM.pdf]

SUPPLEMENTARY INFORMATION

Caldesmon controls stress fiber force-balance through dynamic cross-linking of myosin II and actin-tropomyosin filaments

Shrikant B. Kokate[1], Katarzyna Ciuba[1,5], Vivien Tran[2], Reena Kumari[1], Sari Tojkander[3], Ulrike Engel[4], Konstantin Kogan[1], Sanjay Kumar[2], Pekka Lappalainen[1]*

Contents:

Supplementary Figure 1. Caldesmon and NM-IIA co-localization in contractile stress fibers.

Supplementary Figure 2. The C-terminal domain of Caldesmon localizes to stress fibers, and is critical for stress fiber localization.

Supplementary Figure 3. Interactions of full length Caldesmon and its isolated C-terminal domain of Caldesmon (380-531) with tropomyosin-actin filaments.

Supplementary Figure 4. Confirmation of Caldesmon KO1 and KO2 by Sanger and Next generation sequencing methods.

Supplementary Figure 5. Caldesmon knockout cells display reduced contractility and defects in cell invasion.

Supplementary Figure 6. Effect of Caldesmon deletion on stress fiber mechanics in U2OS cells.

Supplementary Figure 7. Caldesmon depletion does not affect myosin light-chain phosphorylati on or NM-IIA dynamics in cells.

Supplementary Figure 8. Caldesmon deletion affects also α-actinin-1 distribution on stress fibers, but does not disturb tropomyosin organization.

Supplementary Figure 9. Effects of Caldesmon depletion on Tmod1 localization along stress fibers.

Supplementary Figure 10. Capping protein and Caldesmon localizations in wild-type U2OS cells.

Supplementary Figure 11. Smooth muscle specific isoform H-Cald partially rescues the abnormal di stribution of NM-IIA in Caldesmon KO cells.

Supplementary Table 1: Oligonucleotides used in this study

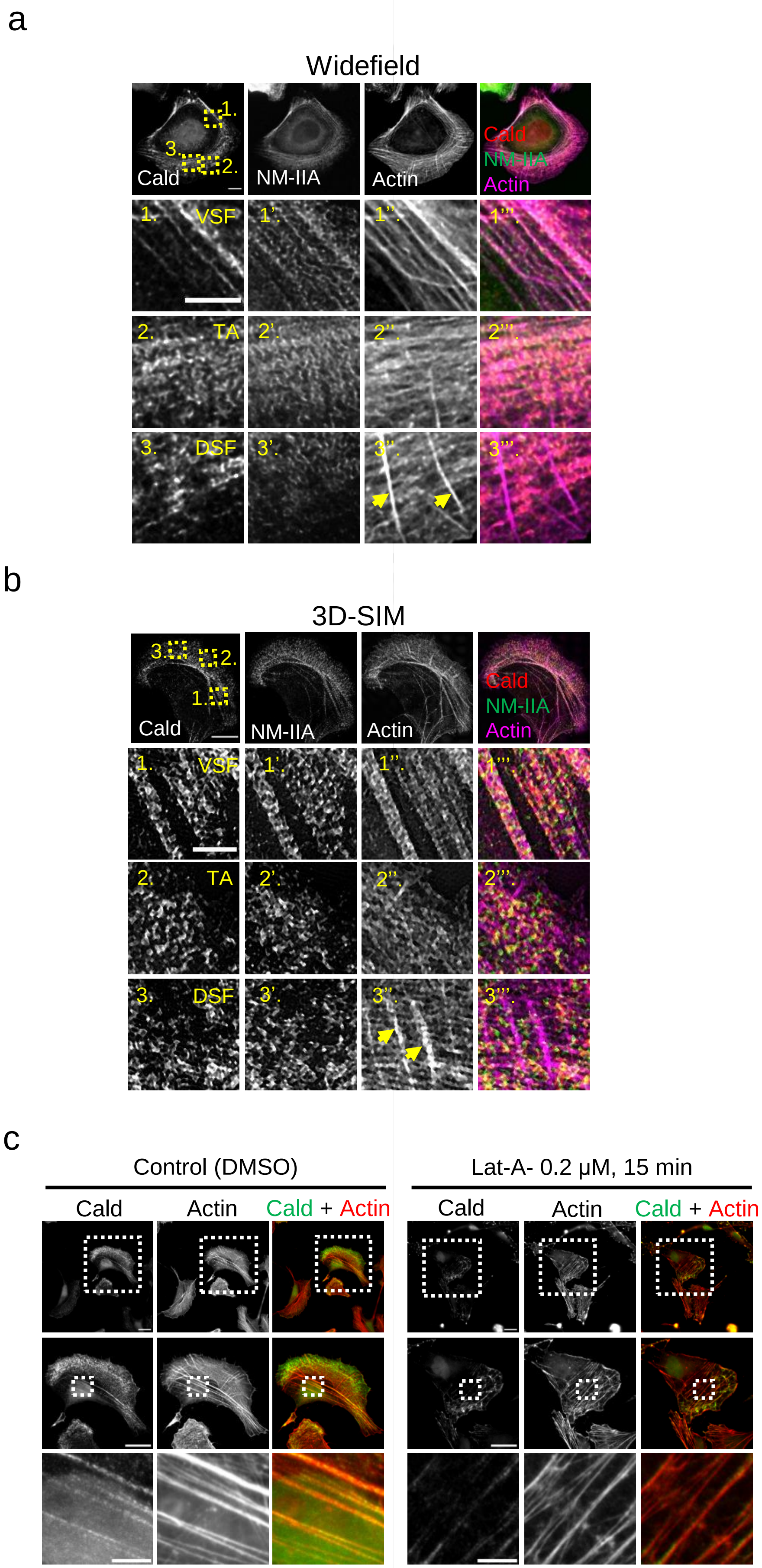
a
Widefield
Cald
NM-IIA
Actin
Cald
NM-IIA
Actin
1.
2.
3.
VSF
1'.
1''.
1'''.
TA
2'.
2''.
2'''.
DSF
3'.
3''.
3'''.
b
3D-SIM
Cald
NM-IIA
Actin
VSF
TA
DSF
c
Control (DMSO)
Lat-A- 0.2 μM, 15 min
Cald
Actin
Cald + Actin

Supplementary Figure 1. Caldesmon and NM-IIA co-localization in contractile stress fibers. Representative examples of Caldesmon, and NM-IIA co-localization in contractile ventral stress fibers (VSF) and transverse arcs (TA) as displayed by wide-field imaging **(a)** and structured illumination super resolution microscopy (3D-SIM) **(b)** approaches. On the other hand, Caldesmon and NM-IIA were not enriched in non-contractile dorsal stress fibers (DFS) indicated by arrows. F-actin was visualized by fluorescent phalloidin. Panels at the bottom are magnifications of the regions indicated with white boxes in the upper panels. Scale bars, whole cell images 10 µm and magnified images 2 µm. **(c)** Representative wide-field imaging examples of wild-type U2OS cells treated with DMSO or DMSO + 0.2 µM Lat-A for 15 mins. Caldesmon and F-actin were visualized by a specific antibody and phalloidin, respectively. Magnified regions (white boxes) illustrate the localization patterns observed for Caldesmon following DMSO and DMSO + Lat-A treatment. Scale bars, 20 µm (top and middle row) and 5 µm (bottom row).

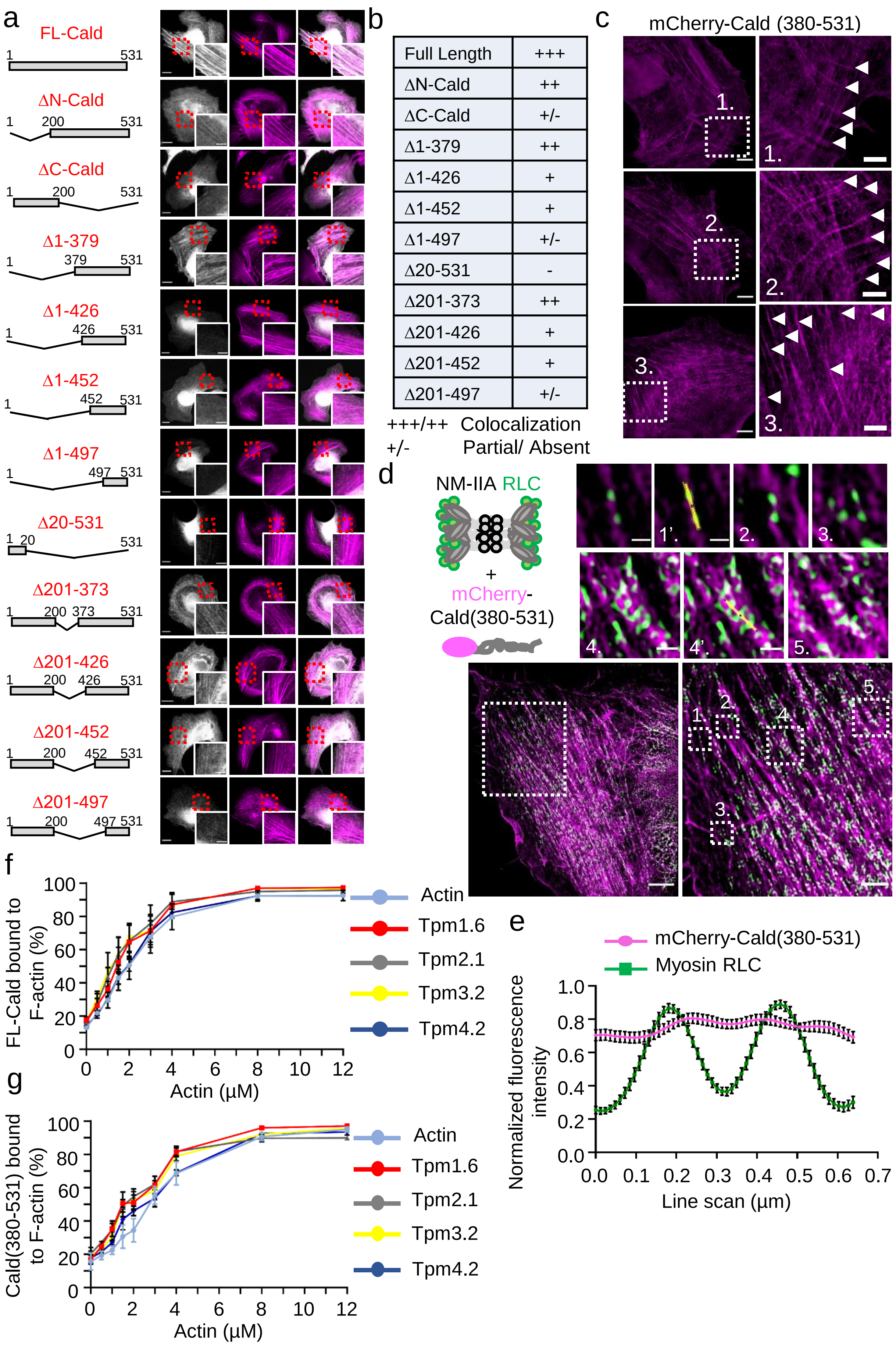

a
FL-Cald
ΔN-Cald
ΔC-Cald
Δ1-379
Δ1-426
Δ1-452
Δ1-497
Δ20-531
Δ201-373
Δ201-426
Δ201-452
Δ201-497
b
Full Length +++
ΔN-Cald ++
ΔC-Cald +/-
Δ1-379 ++
Δ1-426 +
Δ1-452 +
Δ1-497 +/-
Δ20-531 -
Δ201-373 ++
Δ201-426 +
Δ201-452 +
Δ201-497 +/-
+++/++ Colocalization
+/- Partial/ Absent
c
mCherry-Cald (380-531)
d
NM-IIA RLC
+
mCherry-Cald(380-531)
e
mCherry-Cald(380-531)
Myosin RLC
Normalized fluorescence intensity
Line scan (μm)
f
FL-Cald bound to F-actin (%)
Actin (μM)
Actin
Tpm1.6
Tpm2.1
Tpm3.2
Tpm4.2
g
Cald(380-531) bound to F-actin (%)
Actin (μM)

Supplementary Figure 2. The C-terminal domain of Caldesmon localizes to stress fibers, and is critical for stress fiber localization. (a) Representative wide-field microscopy images of U2OS cells transfected with different mCherry-Caldesmon constructs to reveal the contribution of different Caldesmon regions on the localization to stress fibers. mCherry-fusions of full-length-Cald, Cald (201-531), Cald (1-200), Cald (380-531), Cald (427-531), Cald (453-531), Cald (497-531), Cald (1-20) Cald (1-200/374-531), Cald (1-200/427-531), Cald (1-200/453-531) and Cald (1-200/497-531) are in white, and F-actin visualized with Alexa-flour phalloidin-647 is in magenta. Magnified images of the boxed regions show Caldesmon localization to stress fibers. Scale bars, 10 µm (cells), 2 µm (magnified images). **(b)** Table summarizing the localization of Caldesmon truncations to stress fibers. The localization intensities were classified from strong stress fiber localization (+++) to no detectable enrichment to stress fibers (-). The images and analysis are from three experimental repeats. **(c)** Representative examples of 3D-SIM images obtained from cells expressing the mCherry-Caldesmon (380-531) construct. Magnified images illustrate the localization of Caldesmon (380-531) also to dorsal stress fibers (white arrowheads). Scale bars, 5 µm (cells) and 0.5 µm (magnified panels). **(d)** Representative 3D-SIM image from a U2OS cell expressing mCherry-Caldesmon (380-531) construct and stained with an antibody specific to NM-IIA RLC. Magnified regions show myosin II filaments and stacks, as well as relatively uniform localization of mCherry-Caldesmon (380-531) along stress fibers. Scale bars, 5 µm, 2.5 µm, and 1 µm in left, center and right, respectively. **(e)** Line-scans representing fluorescence intensity of NM-IIA RLC and mCherry-Cald(380-531). The data are mean ± S.E.M. of n = 25 filaments from 3 cells. **(f-g)** Co-sedimentation assay to measure binding of full-length Caldesmon (panel F) and its C-terminal region (panel G) to bare β/γ-actin filaments and β/γ-actin filaments saturated with tropomyosins Tpm1.6, Tpm2.1, Tpm3.2, and Tpm4.2. The concentration of Caldesmon constructs was 1 µM. Data are from three experiments, and error bars represent ± S.E.M. Full-length Caldesmon binds F-actin with slightly higher affinity compared to the C-terminal region [Cald (380-531)]. Moreover, saturation of actin filaments with Tpm 1.6, 2.1 and 3.2 appears to enhance their interaction with Caldesmon.

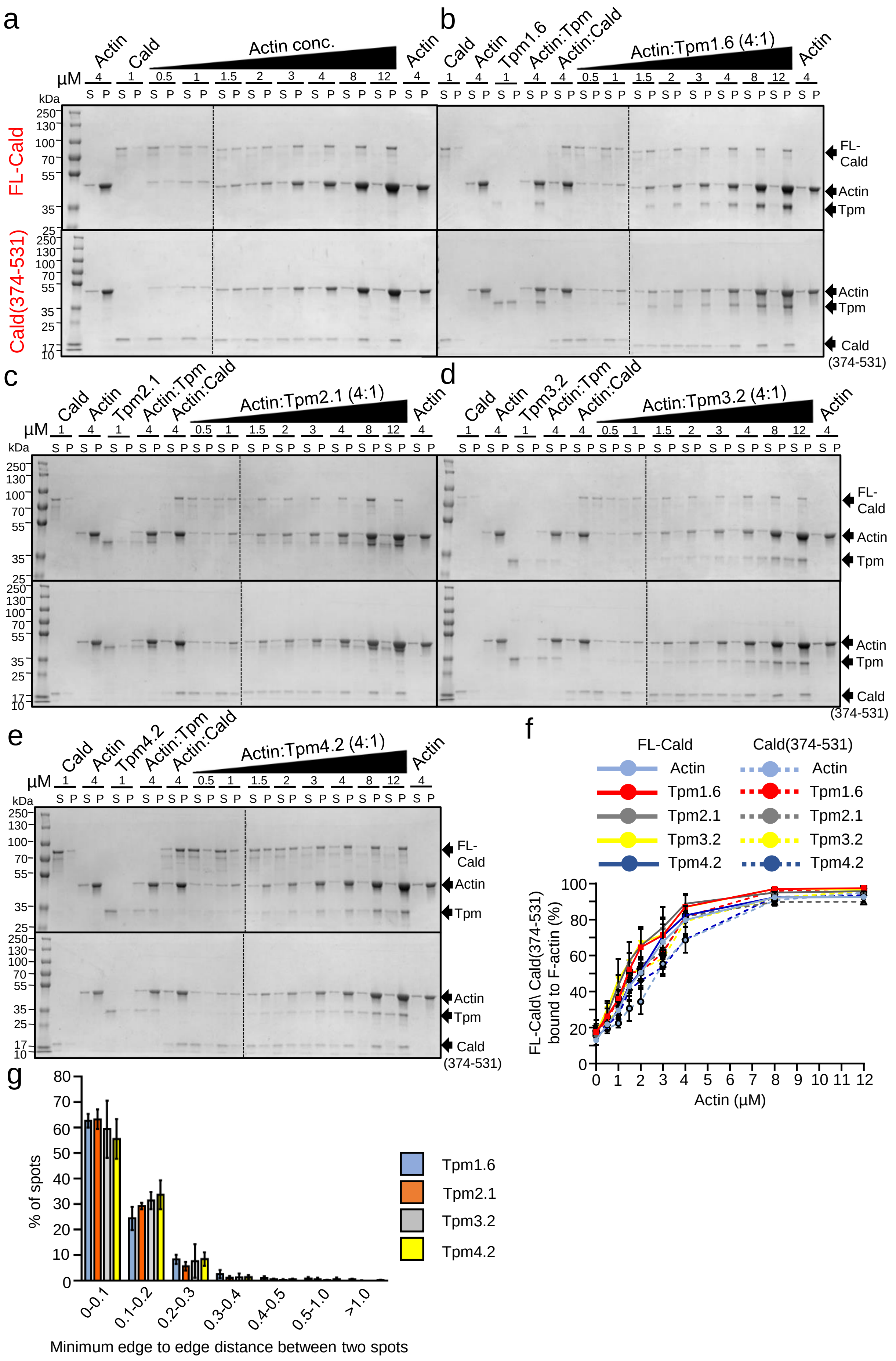

a
Actin
Cald
Actin conc.
µM
4
1
0.5
1
1.5
2
3
4
8
12
S P
kDa
250
130
100
70
55
35
25
17
10
FL-Cald
Cald(374-531)
b
Tpm1.6
Actin:Tpm
Actin:Cald
Actin:Tpm1.6 (4:1)
FL-Cald
Actin
Tpm
Cald (374-531)
c
Tpm2.1
Actin:Tpm2.1 (4:1)
d
Tpm3.2
Actin:Tpm3.2 (4:1)
e
Tpm4.2
Actin:Tpm4.2 (4:1)
f
FL-Cald
Cald(374-531)
Actin
Tpm1.6
Tpm2.1
Tpm3.2
Tpm4.2
FL-Cald\ Cald(374-531) bound to F-actin (%)
Actin (µM)
0
20
40
60
80
100
0 1 2 3 4 5 6 7 8 9 10 11 12
g
% of spots
0
10
20
30
40
50
60
70
80
0-0.1
0.1-0.2
0.2-0.3
0.3-0.4
0.4-0.5
0.5-1.0
>1.0
Minimum edge to edge distance between two spots
Tpm1.6
Tpm2.1
Tpm3.2
Tpm4.2

Supplementary Figure 3. Interactions of full length Caldesmon and its isolated C-terminal domain of Caldesmon (380-531) with tropomyosin-actin filaments. (a-e) Representative gel images from a ctin filament co-sedimentation assays. The upper rows of gel images are from experiments done wi th full-length Caldesmon, and the lower rows are form experiments with Caldesmon (380-531) . **(f)** A graph summarizing the data from all co-sedimentation assays. The amounts (μM) of FL-Cald / Cald (380-531) in pellet fractions (Y-axis) in respect to concentration (μM) of actin/actin:Tpm (X -axis) are shown. Bold lines indicate full-length Caldesmon and dashed lines Cald (380-531). Values = mean; error bars ± S.E.M (n=3). **(g)** 3D-SIM analysis of the co-localization of C-terminus of Caldes mon (using Caldesmon-GFP construct) with Ruby fusions of different tropomyosin isoforms in U2OS cells. Graphical representation shows the minimum distance between C-terminal GFP of Cal desmon with Tpm1.6, Tpm2.1, Tpm3.2 and Tpm4.2 foci along stress fibers. Percentage of (%) Tpm s pots on Y-axis vs. minimum distance between Tpms and Caldesmon C-terminal domain on X-axis (μ m) is shown. Spots analyzed: Tpm1.6 = 115,102 spots (n = 5 cells), Tpm2.1 = 94,220 spots (n = 5 cell s), Tpm3.2 = 144,410 spots (n = 5 cells), Tpm4.2 = 130,660 spots (n = 5 cells). The data represents m ean ± S.D. with 95% confidence interval. Source data are provided as a Source Data file.

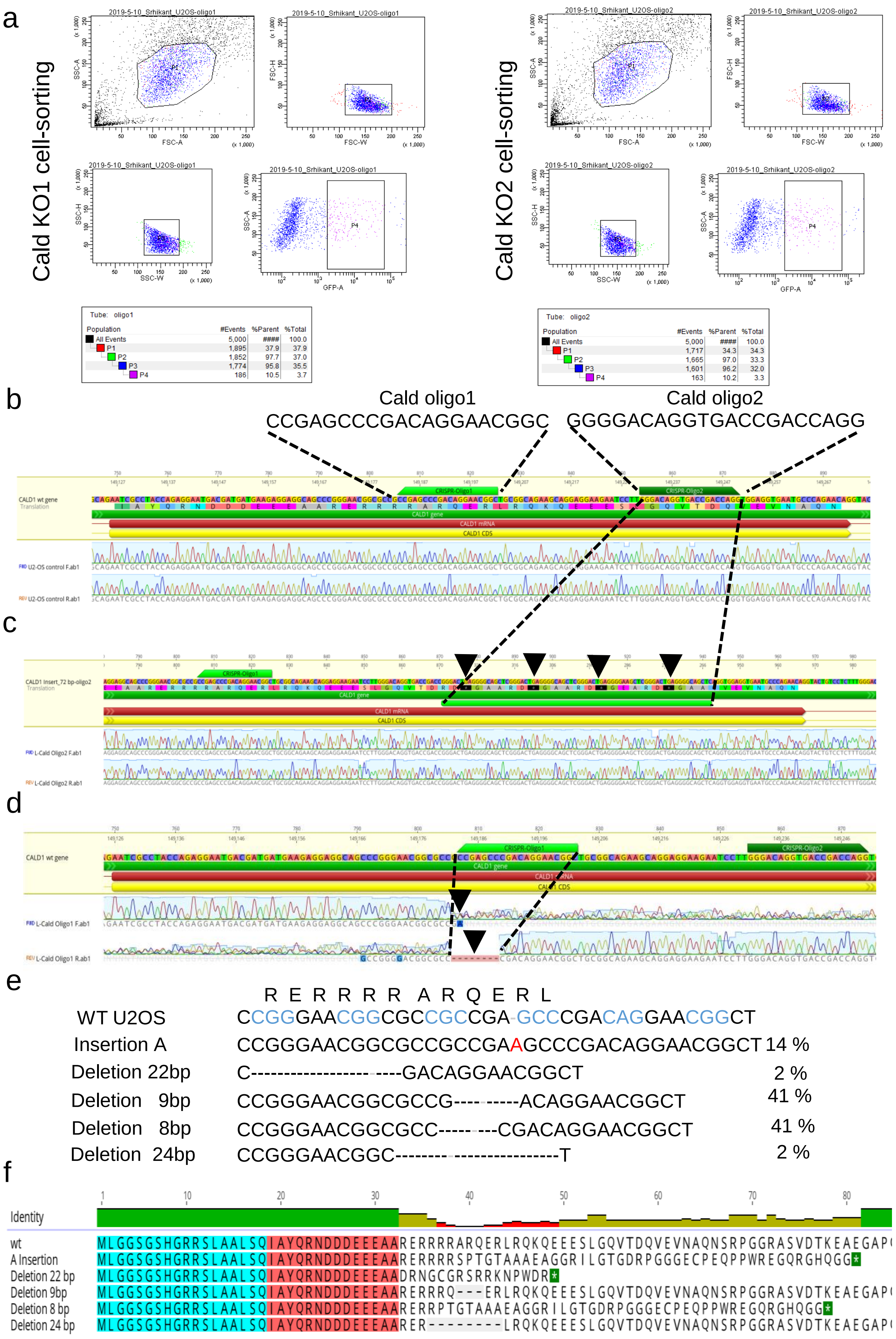
a
Cald KO1 cell-sorting
Cald KO2 cell-sorting
2019-5-10_Srhikant_U2OS-oligo1
2019-5-10_Srhikant_U2OS-oligo2
SSC-A
FSC-A
FSC-H
FSC-W
SSC-H
SSC-W
GFP-A
P1
P4
(x 1,000)
Tube: oligo1
Population | #Events | %Parent | %Total
All Events | 5,000 | #### | 100.0
P1 | 1,895 | 37.9 | 37.9
P2 | 1,852 | 97.7 | 37.0
P3 | 1,774 | 95.8 | 35.5
P4 | 186 | 10.5 | 3.7
Tube: oligo2
Population | #Events | %Parent | %Total
All Events | 5,000 | #### | 100.0
P1 | 1,717 | 34.3 | 34.3
P2 | 1,665 | 97.0 | 33.3
P3 | 1,601 | 96.2 | 32.0
P4 | 163 | 10.2 | 3.3
b
Cald oligo1
CCGAGCCCGACAGGAACGGC
Cald oligo2
GGGGACAGGTGACCGACCAGG
CALD1 wt gene
CRISPR-Oligo1
CRISPR-Oligo2
CALD1 gene
CALD1 mRNA
CALD1 CDS
U2-OS control F.ab1
U2-OS control R.ab1
c
CALD1 Insert_72 bp-oligo2
L-Cald Oligo2 F.ab1
L-Cald Oligo2 R.ab1
d
L-Cald Oligo1 F.ab1
L-Cald Oligo1 R.ab1
e
R E R R R R A R Q E R L
WT U2OS CCGGGAACGGCGCCGCCGA-GCCCGACAGGAACGGCT
Insertion A CCGGGAACGGCGCCGCCGAAGCCCGACAGGAACGGCT 14 %
Deletion 22bp C----------------------GACAGGAACGGCT 2 %
Deletion 9bp CCGGGAACGGCGCCG---------ACAGGAACGGCT 41 %
Deletion 8bp CCGGGAACGGCGCC--------CGACAGGAACGGCT 41 %
Deletion 24bp CCGGGAACGGC-------------------------T 2 %
f
Identity
wt MLGGSGSHGRRSLAALSQIAYQRNDDDEEEAARERRRRARQERLRQKQEEESLGQVTDQVEVNAQNSRPGGRASVDTKEAEGAP
A Insertion MLGGSGSHGRRSLAALSQIAYQRNDDDEEEAARERRRRSPTGTAAAEAGGRILGTGDRPGGGECPEQPPWREGQRGHQGG*
Deletion 22 bp MLGGSGSHGRRSLAALSQIAYQRNDDDEEEAADRNGCGRSRRKNPWDR*
Deletion 9bp MLGGSGSHGRRSLAALSQIAYQRNDDDEEEAARERRRQ---ERLRQKQEEESLGQVTDQVEVNAQNSRPGGRASVDTKEAEGAP
Deletion 8 bp MLGGSGSHGRRSLAALSQIAYQRNDDDEEEAARERRPTGTAAAEAGGRILGTGDRPGGGECPEQPPWREGQRGHQGG*
Deletion 24 bp MLGGSGSHGRRSLAALSQIAYQRNDDDEEEAARER--------LRQKQEEESLGQVTDQVEVNAQNSRPGGRASVDTKEAEGAP

Supplementary Figure 4. Confirmation of Caldesmon KO1 and KO2 by Sanger and Next generation sequencing methods. **(a)** Representative images of Caldesmon KO1 and KO2 cells sorting by FACS Aria-II machine. **(b-d)** Representative results showing DNA sequences obtained from wild-type (panel B), Caldesmon KO2 (panel C) and Caldesmon KO1 (panel D) clones generated by CRISPR-Cas9 approach. Guide RNA sequences that were targeted to *Caldesmon* exon 1 to generate KO1 (fluorescent green) and KO2 (dark green) are shown above the sequences. **(e)** Next generation sequencing performed to confirm Caldesmon KO-oligo1 mutation revealed that the Cald KO1 cells had a heterogeneous population of different mutations with Adenine insertion (14%), 22 bp deletion (2%), 24 bp deletion (2%), 9 bp deletion (41%) and 8 bp deletion (41%) respectively. Thus, these appear to represent a mixture of at least two different cell populations, where Caldesmon alleles are disrupted by different ways. Importantly, no wild-type *Caldesmon* was detected in the NGS analysis. **(f)** Sequence alignment of all the NGS sequences of the KO1 clone with wild-type sequence confirmed that guide RNA 1 led to generation of different truncations in Caldesmon KO1 cells.

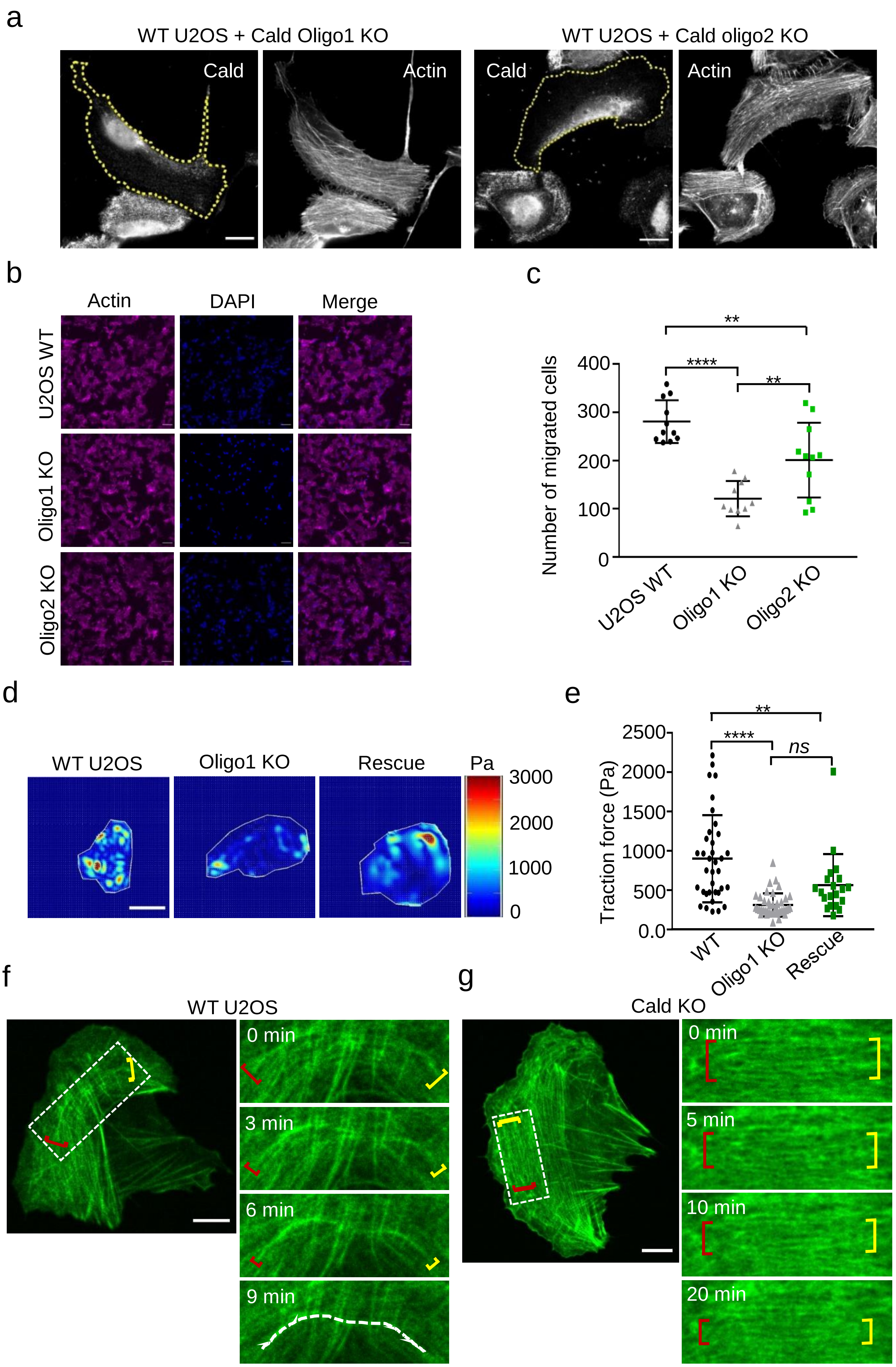

a
WT U2OS + Cald Oligo1 KO
Cald
Actin
WT U2OS + Cald oligo2 KO
Cald
Actin
b
Actin
DAPI
Merge
U2OS WT
Oligo1 KO
Oligo2 KO
c
Number of migrated cells
400
300
200
100
0
**
****
**
U2OS WT
Oligo1 KO
Oligo2 KO
d
WT U2OS
Oligo1 KO
Rescue
Pa
3000
2000
1000
0
e
Traction force (Pa)
2500
2000
1500
1000
500
0.0
**
****
ns
WT
Oligo1 KO
Rescue
f
WT U2OS
0 min
3 min
6 min
9 min
g
Cald KO
0 min
5 min
10 min
20 min

Supplementary Figure 5. Caldesmon knockout cells display reduced contractility and defects in ce ll invasion. (a) Representative wide-field images of Cald oligo1 and oligo2 knockout U2OS cells that were mixed wild-type U2OS cells, and stained with Caldesmon-specific antibody and phalloidin. Cal desmon-depleted cells (indicated by dotted lines) display less organized stress fiber network comp ared to wild-type cells, and display abnormal morphology and sizes. Scale bars, 15 µm. The images are representatives of three experiments. **(b)** 3D-Matrigel invasion assay image of wild-type, Caldes mon KO1 and Caldesmon KO2 cells, showing cells that passed through the matrigel. F-actin was vis ualized by alexa-flour-phalloidin 647 and cell-nuclei were stained by DAPI. Scale bars, 50 µm. **(c)** Corresponding scatter plot representing the numbers of wild-type, Caldesmon KO1 and Cal desmon KO2 cells invaded through the matrigel. Data points represent the mean number of cells fr om at least three different view fields from three independent experiments. The data represent m ean ± S.D. (Un-paired t-test showing two-tailed p-values with 95% confidence interval). *p*-values: *p*-values = ns ($p = 0.1234$); ** ($p = 0.0067$: U2OS WT vs Oligo 2 KO, $p = 0.0079$: Oligo1 KO vs Oligo2 KO); **** ($p < 0.0001$). **(d)** Representative Force maps of wild-type, Cald KO1 and KO1-rescue cells see ded on 26 kPa polyacrylamide substrates with 488-labelled nanobeads. Scale bars, 20 µm. **(e)** Quan tification of traction forces as RMS values from wild-type, Caldesmon KO1 and Caldesmon KO1-resc ue cells. Graph represents mean ± S.D. from n = 36 (wild-type), n = 40 (KO1) and n = 20 (KO1-rescue) for individual cells from three experimental repeats. One-way ANOVA with Tukey's mul tiple comparison test. *p*-values: ns ($p = 0.0531$); ** ($p = 0.0088$); **** ($p < 0.0001$). The same values for wild-type cells are also represented in figure 4B. **(f-g)** Live-cell imaging of wild-type (panel F) an d Caldesmon KO2 cells (panel G) transfected with GFP-Lifeact demonstrating that in a wild-type cell transverse arcs display centripetal flow (indicated by red and yellow brackets), and eventually fuse with each other to form a thick actomyosin bundle (white dashed line). Transverse arc flow in the C aldesmon KO2 cells is slower and less uniform. Scale bars = 15 µm (cells on the left), 5 µm (magnifications). Source data are provided as a Source Data file.

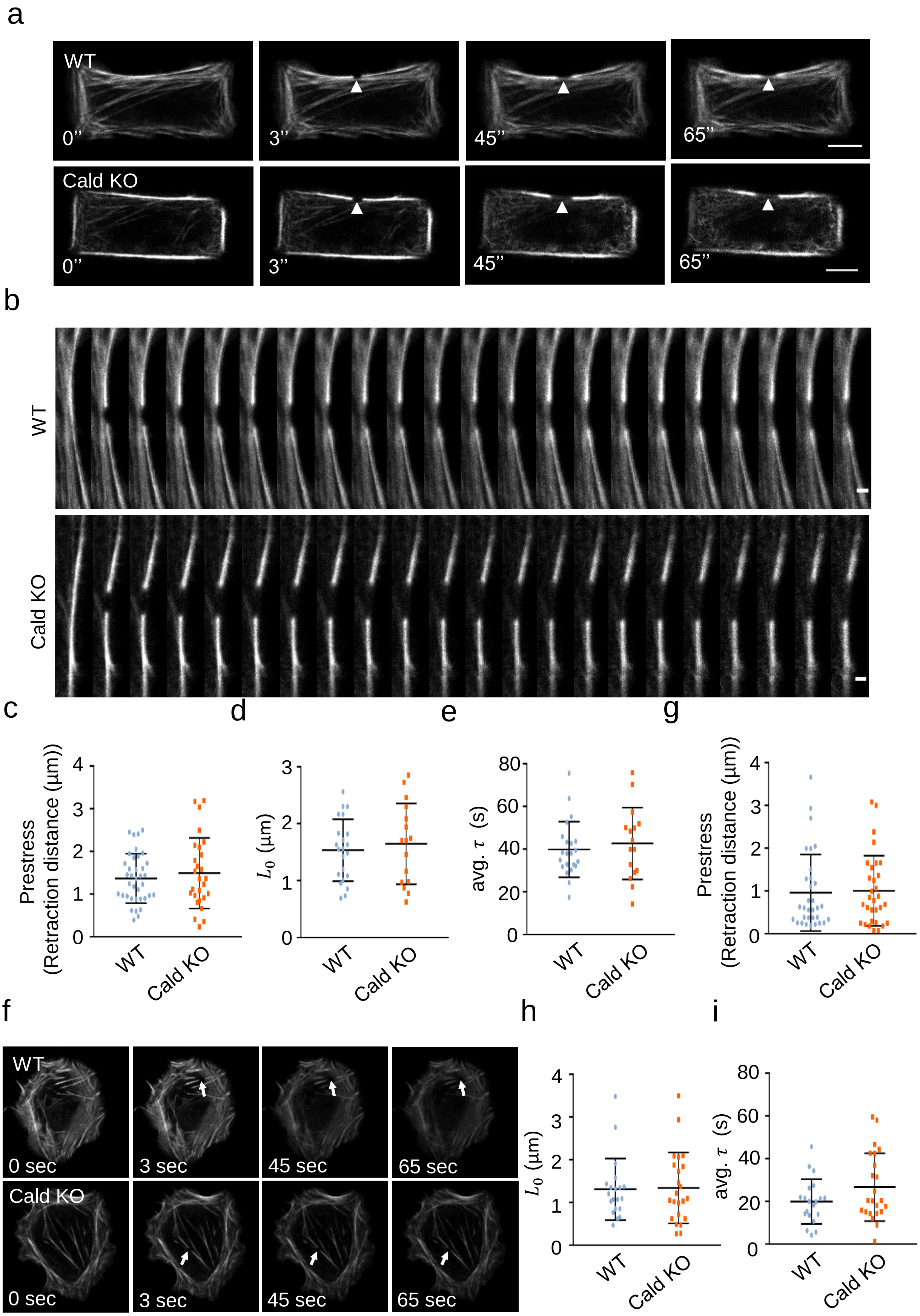
a
WT
0''
3''
45''
65''
Cald KO
0''
3''
45''
65''
b
WT
Cald KO
c
Prestress
(Retraction distance (µm))
WT
Cald KO
d
L_0 (µm)
WT
Cald KO
e
avg. τ (s)
WT
Cald KO
g
Prestress
(Retraction distance (µm))
WT
Cald KO
f
WT
0 sec
3 sec
45 sec
65 sec
Cald KO
0 sec
3 sec
45 sec
65 sec
h
L_0 (µm)
WT
Cald KO
i
avg. τ (s)
WT
Cald KO

Supplementary Figure 6. Effect of Caldesmon deletion on stress fiber mechanics in U2OS cells. (a) Representative images of laser ablation experiment performed using wild-type and Caldesmon KO2 U2OS cells grown on U-shaped micropatterns. Cells at 0 s illustrate the distribution of SFs pre-ablati on, and cells at 3 s illustrate the distribution of SFs immediately after ablation. White arrows highlig ht the retraction of the stress fiber post-ablation at 3 s, 45 s and 65 s from start of imaging. Scale b ars = 10 µm. **(b)** Kymograph of the severed SFs in Figure A, with 3 s between slices and 65 s elapsed . Scale bars = 2 µm. **(c)** Model-independent ventral SF retraction analysis of patterned wild-ty pe and Caldesmon KO U2OS cells. The distribution of the retraction at t=60 s post-ablation of one c ut end of the stress fiber of both wild-type and KO cells. The data from C. represents mean value ± S.D. from n = 37 (wild-type) and n = 27 (Caldesmon KO) from three experimental replicates. Differe nces are non-significant (Mann-Whitney U test). **(d)** Plateau retraction distance (L_o) and **(e**) viscoela stic time constant (τ) for severed SFs in patterned wild-type and Caldesmon KO U2OS cells. The dat a from D. and E. represent mean value ± S.D. from n = 22 (wild-type) and n = 16 (Caldesmon KO) cel ls from four experimental replicates. Non-KV retractions were excluded from the dataset of D. and E. Differences are non-significant (Mann-Whitney U test). **(f)** Representative images of laser ablatio n experiment performed using wild-type and Caldesmon KO U2OS cells grown on unpatterned glas s. Cells at 0 s illustrate the distribution of SFs pre-ablation, and cells at 3 s illustrate the distribution of SFs immediately after ablation. White arrows highlight the retraction of the SF post-ablation at 3 s, 45 s and 65 s from start of imaging. Scale bars = 10 µm. **(g)** Model-independent SF retraction anal ysis of unpatterned wild-type and Caldesmon KO U2OS cells. The distribution of the retraction at t= 60 s post-ablation of one cut end of the stress fiber for wild-type and KO cells. The data from G. rep resent mean value ± S.D. from n = 32 (wild-type) and n = 31 (Caldesmon KO) from three experimen tal replicates. Differences are non-significant (two-tailed, non-parametric Mann-Whitney U test). **(h)** Plateau retraction distance (L_o) and **(i)** viscoelastic time constant (τ) for severed SFs in patterned wild-type and Caldesmon KO U2OS cells. The data from H. and I. represents mean value ± S.D. from n = 20 (wild-type) and n = 23 (Caldesmon KO) cells from three experimental replicates. Non-KV retr actions were excluded from the dataset of H. and I. Differences are non-significant (two-tailed, non -parametric Mann-Whitney U test). Source data are provided as a Source Data file.

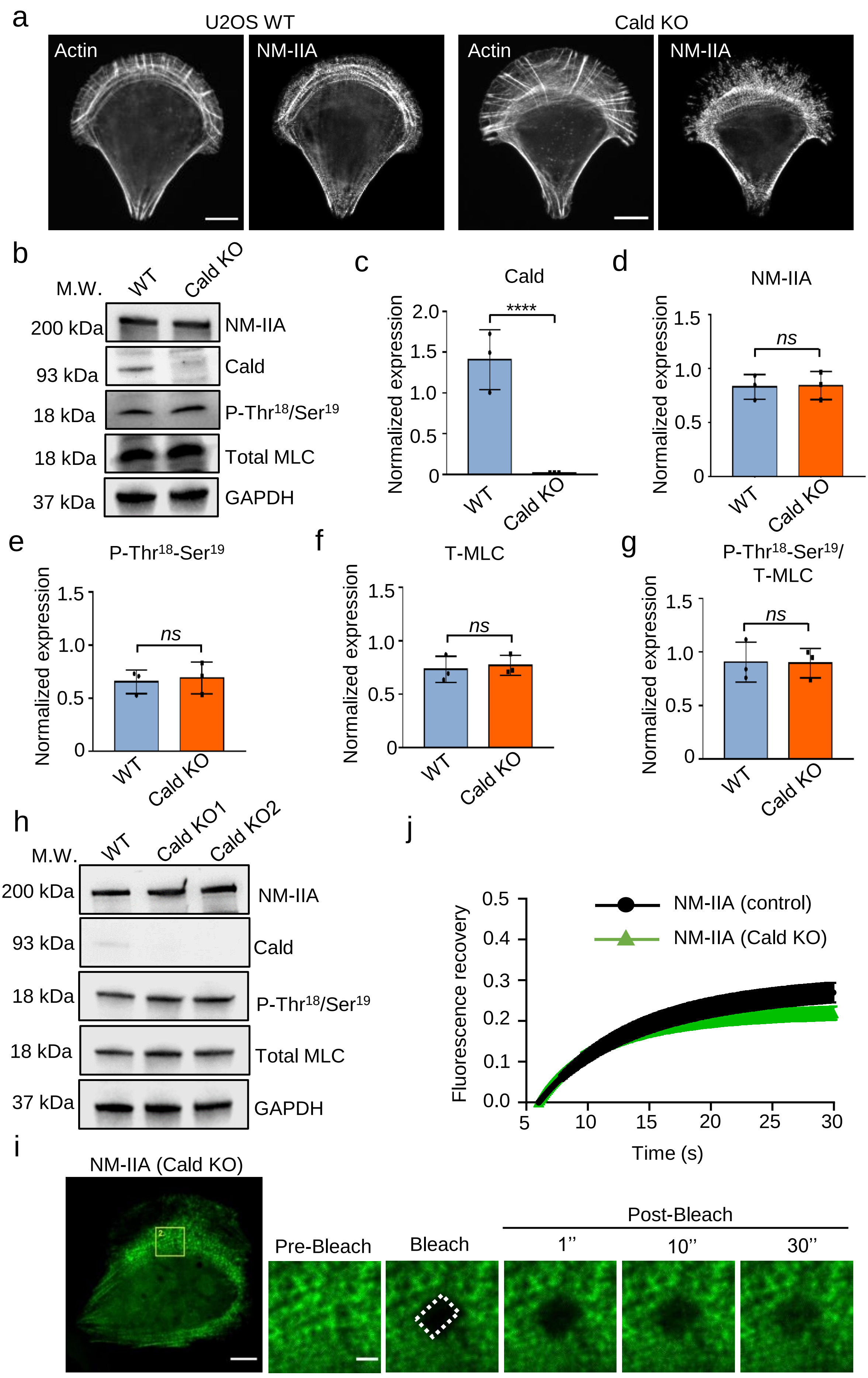

a
U2OS WT
Cald KO
Actin
NM-IIA
b
M.W.
WT
Cald KO
200 kDa
NM-IIA
93 kDa
Cald
18 kDa
P-Thr18/Ser19
18 kDa
Total MLC
37 kDa
GAPDH
c
Cald
Normalized expression
****
d
NM-IIA
ns
e
P-Thr18-Ser19
f
T-MLC
g
P-Thr18-Ser19/
T-MLC
h
Cald KO1
Cald KO2
j
Fluorescence recovery
NM-IIA (control)
NM-IIA (Cald KO)
Time (s)
i
NM-IIA (Cald KO)
Pre-Bleach
Bleach
Post-Bleach
1''
10''
30''

Supplementary Figure 7. Caldesmon depletion does not affect myosin light-chain phosphorylation or NM-IIA dynamics in cells. (a) Representative wide-field images of wild-type and Cald oligo2 knockout U2OS cells grown on crossbow-shaped micropatterns, and stained with NM-IIA specific antibodies and phalloidin. Caldesmon knockout cells (right) display an irregular stress fiber network, and disturbed NM-IIA distribution along cell periphery. Scale bars, 10 µm. The images are representatives of three experiments. **(b)** Representative example of a Western blot performed using whole cell lysates of

wild-type and Cald KO2 cells. The blots revealed that NM-IIA, MLC and Thr^{18}/Ser^{19}-phosphorylation levels remained unaltered in Caldesmon knockout cells. GAPDH was probed to detect equal protein loading. Densitometric quantifications showing mean ± S.D. of normalized protein expressions of Caldesmon **(c),** NM-IIA **(d),** Phospho-MLC **(e),** Total MLC **(f),** and Phospho-MLC/ Total MLC ratio **(g)** from three independent Western blots. Two-tailed unpaired t-test with Welch's correction. *p*-values: ns ($p = 0.1234$); **** ($p < 0.0001$) **(h)** Western blot experiment performed using whole cell lysates from wild-type, Caldesmon KO1 and Caldesmon KO2 cells showing protein levels of Caldesmon, NM-IIA, Total-MLC, Phospho-MLC and GAPDH. The blots summarize observations from three experimental repeats. **(i)** Representative example of a FRAP experiment performed on GFP-NM-IIA expressing Caldesmon KO cells. The white box in magnified image represents the region where the rate of NM-IIA photo recovery was measured. Scale bars, 15 µm (cell), 5 µM (magnified images). **(j)** Graphical representation showing FRAP recovery curves of NM-IIA heavy chain. The graphical data represents mean ±S.E.M. of recovery curves in wild-type (n = 23) and Caldesmon KO (n = 21) cells. The same NM-IIA curve of wild-type cells is also shown in Figure 1E. Source data are provided as a Source Data file.

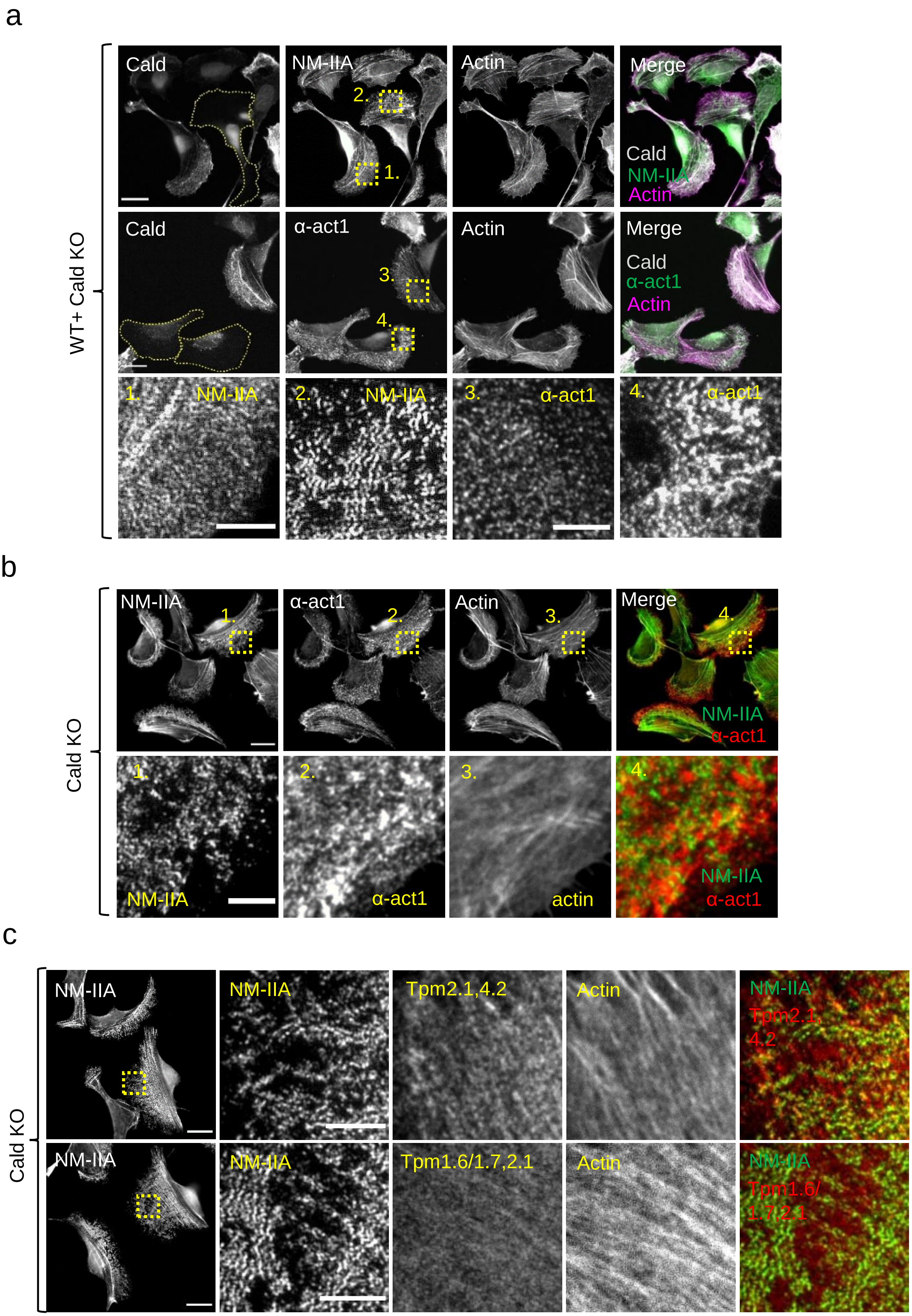
a
WT+ Cald KO
Cald
NM-IIA
Actin
Merge
Cald
NM-IIA
Actin
2.
1.
Cald
α-act1
Actin
Merge
Cald
α-act1
Actin
3.
4.
1. NM-IIA
2. NM-IIA
3. α-act1
4. α-act1
b
Cald KO
NM-IIA
α-act1
Actin
Merge
1.
2.
3.
4.
NM-IIA
α-act1
1.
2.
3.
4.
NM-IIA
α-act1
actin
NM-IIA
α-act1
c
Cald KO
NM-IIA
NM-IIA
Tpm2.1,4.2
Actin
NM-IIA
Tpm2.1,
4.2
NM-IIA
NM-IIA
Tpm1.6/1.7,2.1
Actin
NM-IIA
Tpm1.6/
1.7,2.1

Supplementary Figure 8. Caldesmon deletion affects also α-actinin-1 distribution on stress fibers, but does not disturb tropomyosin organization. (a) Representative examples of wild-type and Caldesmon KO cells mixed with each other, and stained with antibodies against Caldesmon, and NM-IIA (upper row), and Caldesmon and α-actinin-1 (middle row), as well as with fluorescent phalloidin to visualize F-actin. The Caldesmon knockout cells are indicated with dashed lines. The magnified images on the bottom row (from boxed areas indicated above) demonstrate regular NM-IIA localization along stress fibers of wild-type cell (1), irregular NM-IIA distribution in Caldesmon knockout cells (2), regular α-actinin-1 localization in wild-type cell (3) and irregular α-actinin-1 localization in Caldesmon KO cell (4). **(b)** Caldesmon KO cells stained with antibodies against NM-IIA and α-actinin-1. F-actin was visualized by phalloidin. The two magnified images of α-actinin-1 and NM-IIA staining (right) demonstrate that the α-actinin-1 and NM-IIA localizations are differentially disrupted, and they do not co-localize with each other on stress fibers. **(c)** Localizations of NM-IIA (visualized by NM-IIA coiled coil-specific antibody), Tpm 2.1/4.2 (top row, visualized by LC24 antibody), and Tpm1.6/1.7/2.1 (bottom row, visualized by TM311) in Caldesmon KO cells. F-actin was visualized by fluorescent-phalloidin. Magnified images on the right (from the areas indicated by dashed boxes in the whole cell images) indicate that the regular localization of tropomyosins was not disrupted in Caldesmon KO cells, despite the irregular NM-IIA pattern. Scale bars, cells 20 µm; magnified images 5 µm.

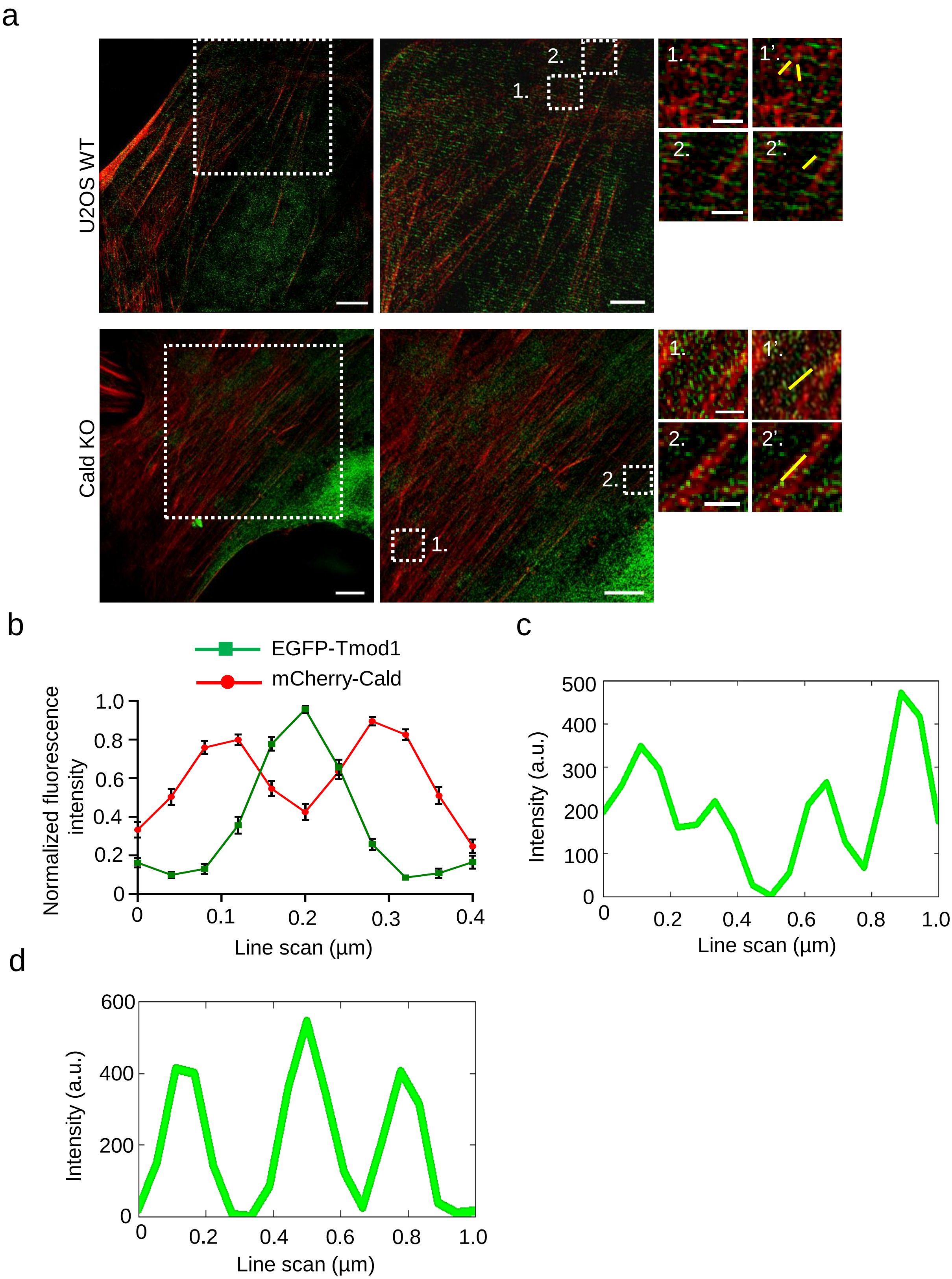

a
U2OS WT
Cald KO
1.
2.
1'.
2'.
b
EGFP-Tmod1
mCherry-Cald
Normalized fluorescence intensity
1.0
0.8
0.6
0.4
0.2
0
0.1
0.2
0.3
0.4
Line scan (µm)
c
Intensity (a.u.)
500
400
300
200
100
0
0.2
0.4
0.6
0.8
1.0
Line scan (µm)
d
Intensity (a.u.)
600
400
200
0
0.2
0.4
0.6
0.8
1.0
Line scan (µm)

Supplementary Figure 9. Effects of Caldesmon depletion on Tmod1 localization along stre ss fibers. (a) Representative SIM examples of a wild-type cell (top row) expressing GFP-Tmod 1 (green) and mCherry-Caldesmon (red), and a Caldesmon KO cell (bottom row) expressing GFP-T mod1 (green). F-actin was visualized in the knockout cell by phalloidin (red). The thin yellow lines in the magnified regions 1’ and 2’ of the wild-type and Caldesmon KO cells illustrate examples of regi ons used for the line scan analysis. Scale bars, 5 µm, 3 µm and 1 µm for the left, middle, and right p anels, respectively. **(b)** Line plot analysis illustrating localization pattern of Caldesmon in respect to Tmod1 in wild-type U2OS cells. Line scans = mean ± S.E.M. of n = 30 filaments from 3 cells. **(c)** and **(d)** Representative individual line scans from a Caldesmon KO cell-(from panel A) demonstratin g irregular Tmod1 localization along stress fibers.

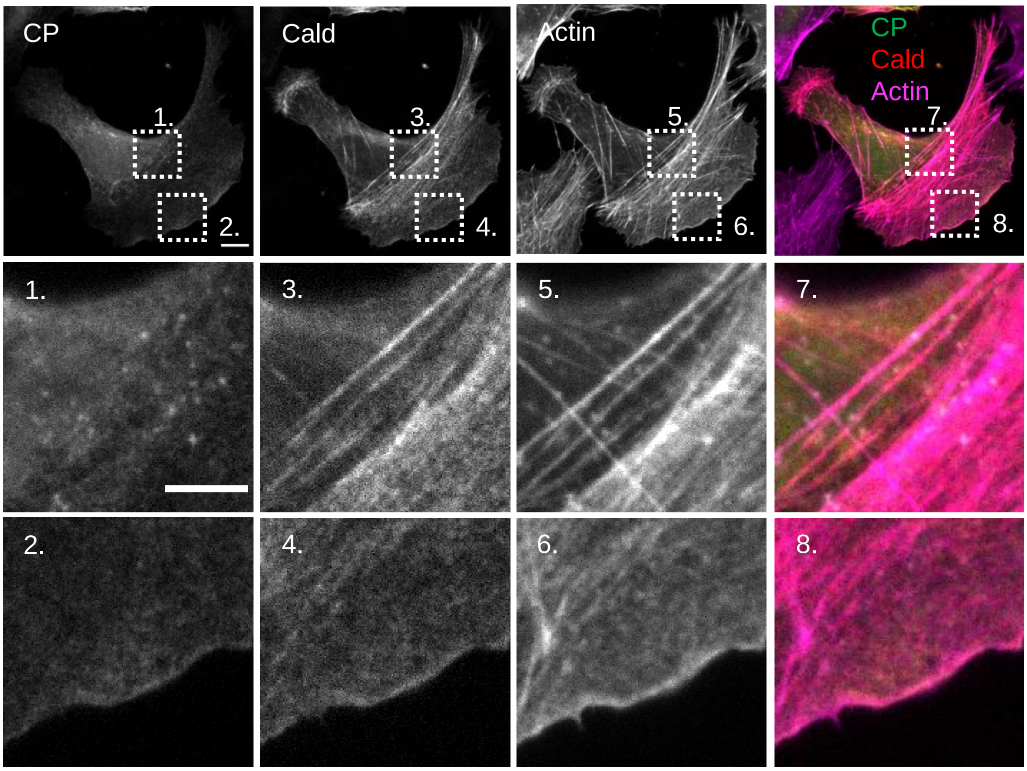

Supplementary Figure 10. Capping protein and Caldesmon localizations in wild-type U2OS cells. R epresentative wide-field images showing localizations of eGFP-CP-β, mCherry-Cald, and F-actin (visualized by phalloidin) in a wild-type U2OS cell. Magnified images 1, 3, 5, and 7 from the cell cen ter demonstrate localization of Caldesmon to thick ventral stress fibers and localization of CP to act in-rich puncta, which most likely represent endocytic invaginations. Magnified regions 2, 4, 6, a nd 8 show CP localization to lamellipodium and Caldesmon enrichment in transverse arcs close to the cell edge. Scale bars 10 µm (whole cell), 2 µm (magnified regions).

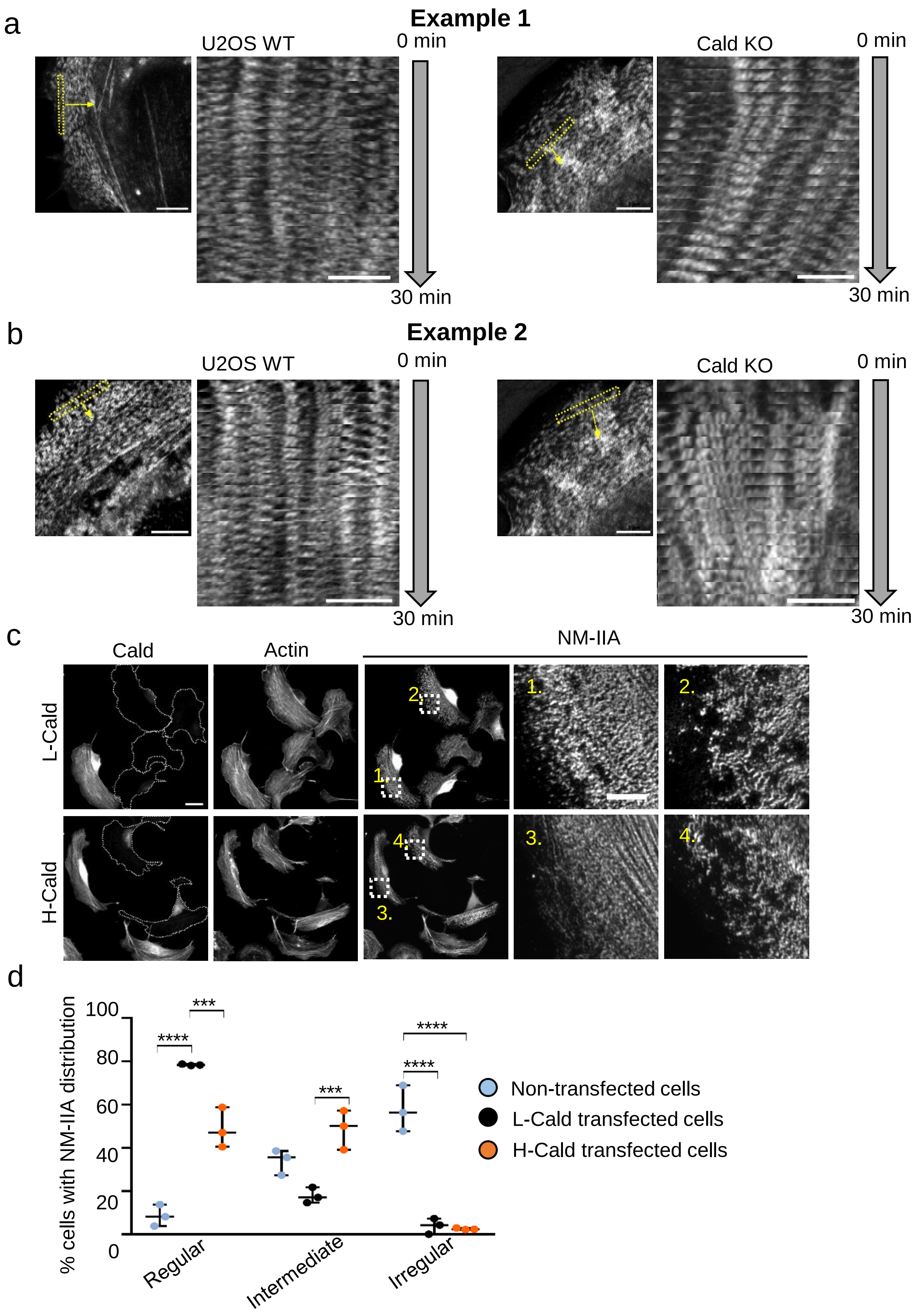
a
Example 1
U2OS WT
0 min
30 min
Cald KO
0 min
30 min
b
Example 2
U2OS WT
0 min
30 min
Cald KO
0 min
30 min
c
Cald
Actin
NM-IIA
L-Cald
H-Cald
1.
2.
3.
4.
d
% cells with NM-IIA distribution
100
80
60
40
20
0
Regular
Intermediate
Irregular
****
***
***
****
****
Non-transfected cells
L-Cald transfected cells
H-Cald transfected cells

Supplementary Figure 11. Smooth muscle specific isoform H-Cald partially rescues the abnormal distribution of NM-IIA in Caldesmon KO cells. (a) and **(b)** Representative examples of kymographs showing fluctuations of myosin filaments within stress fibers undergoing retrograde flow in wild type and Caldesmon KO cells. Whole cell images on the left highlight the stress fibers (indicated by dashed boxes) used for the kymograph analysis, and the thin yellow arrows indicate the direction of myosin filament flow in cells. The grey arrows to the right of kymographs indicate the time scale. Bars, 5 µm (whole cells) and 3 µm (magnified regions). **(c)** Immunofluorescence microscopy examples of Cald oligo2 KO U2OS cells expressing mCherry-L-Cald and mCherry-H-Cald constructs. Cells were stained for myosin coiled-coil and F-actin. Dashed-lines highlight non-transfected Cald KO cells, whereas L-Cald (first row) and H-Cald (second row) transfected cells were detected by mCherry-expression. Magnified images on the right correlate with the dashed boxed, and highlight the myosin filament distributions in the respective cells. Scale bars, 20 µm (whole cells) and 5 µm (magnifications). **(d)** Blind analysis of NM-IIA filament distribution along stress fibers in Caldesmon knockout U2OS cells expressing mCherry-L-Cald or mCherry-H-Cald constructs. Data represent mean ± S.D. (two-way ANOVA followed by Tukey's multiple comparisons test) of non-transfected (n=350), as well as L-Cald (n=134) and H-Cald (n=122) expressing cells from three experiments. Statistical significance: *** (p = 0.0006: L-Cald transfected-regular vs H-Cald transfected-regular, p = 0.0003: L-Cald transfected-intermediate vs H-Cald transfected-intermediate); **** (p < 0.0001). Source data are provided as a Source Data file.

SUPPLEMENTARY TABLE 1: OLIGONUCLEOTIDES USED IN THIS STUDY

Primer type	Primer sequences (5'-3')	Plasmid library no.
**FL-Cald:**		pPL1374
Forward	GTAGTACTCGAGTGACCACCATGCTTAGCAGATCCGGGT	
Reverse	GTAGTAGGATCCTCAGACCTTAGTGGGAGAAGT	
**Cald CRISPR-Cas9 guide 1:**		pPL1899
Forward	CACCGGCCGTTCCTGTCGGGCTCGG	
Reverse	AAACCCGAGCCCGACAGGAACGGCC	
**Cald CRISPR-Cas9 guide 2:**		pPL1900
Forward	CACCGGGGACAGGTGACCGACCAGG	
Reverse	AAACCCTGGTCGGTCACCTGTCCCC	
**Cald KO NGS:**		
Forward	ACACTCTTTCCCTACACGACGCTCTTCCGATCTCAGTTTCCAAAGGCA ACTTGCTTTTGTG	
Reverse	GTGACTGGAGTTCAGACGTGTGCTCTTCCGATCTTGAAATGAAATAAA AGCCTTCAACTGGG	
**Cald KO1 Sanger sequencing:**		
Forward	CACCGGCCGTTCCTGTCGGGCTCGG	
Reverse	GGCCGTTCCTGTCGGGCTCGGGTTT	
**Cald KO2 Sanger sequencing:**		
Forward	CACCGGGGACAGGTGACCGACCAGG	
Reverse	GGGGACAGGTGACCGACCAGGGTTT	
**Cald domain deletions:**		
**mCherry-FL-Cald:**		PL1375
Forward	GTAGTACTCGAGTGACCACCATGCTTAGCAGATCCGGGT	
Reverse	GTAGTAGGATCCTCAGACCTTAGTGGGAGAAGT	
**mCherry-FL-Cald-EGFP:**		pPL1688
Forward (mCherry-Cald)	TACAAGTGATCTAGATAACTGATCATAATCAGCCATACCAC	
Reverse (mCherry-Cald)	AGAGGTGGATCCGCTAGAACCGACCTTAGTGGGAGAAGTGACCT TATC	
Forward (EGFP)	TTCTAGCGGATCCACCTCTGGAATGGTGAGCAAGGGCGAGGAG	
Reverse (EGFP)	TATGATCAGTTATCTAGATCACTTGTACAGCTCGTCCATGCCG	
**FL-Cald-EGFP:**		PL1793
Forward	TCGCCACCATGCTTAGCAGATCCGGGTCCCAG	
Reverse	TGCTAAGCATGGTGGCGACCGGTAGCGCTAG	
**mCherry-Cald(201-531):**		pPL1671

Forward	AGTGACCACCATCAAAGATGAGAAGATTAAAAAGGACAAAGAGCCC	
Reverse	ATCTTTGATGGTGGTCACTCGAGATCTGAGTCCG	
**mCherry-Cald(1-200):**		pPL1672
Forward	CGAAAATCAGTGAGGATCCACCGGATCTAGATAACTGATCA	
Reverse	TGGATCCTCACTGATTTTCGCCAAGGTTCCCTCCCTTC	
**mCherry-Cald(380-531):**		pPL1673
Forward	AGTGACCACCAAGTGCTTCACTCCTAAAGGCTCATCTCTC	
Reverse	CGGACTCAGATCTCGAGTGACCACCAAGTGCTTCA	
**mCherry-Cald(427-531):**		pPL1674
Forward	GTGACCACCACAAAAGCTTCAAAACCTATGAAGCCTGC	
Reverse	GAAGCTTTTGTGGTGGTCACTCGAGATCTGAGTCCG	
**mCherry-Cald(453-531):**		pPL1675
Forward	GTGACCACCATGTGGGAGAAAGGGAGTGTGTTTTCATC	
Reverse	CTCCCACATGGTGGTCACTCGAGATCTGAGTCCG	
**mCherry-Cald(498-531):**		pPL1676
Forward	GTGACCACCCCCGCTCCCAAGCCTTCTGAC	
Reverse	TGGGAGCGGGGGTGGTCACTCGAGATCTGAGTCCG	
**mCherry-Cald(1-19):**		pPL1677
Forward	CTCTCAAATTTGAGGATCCACCGGATCTAGATAACTG	
Reverse	TGGATCCTCAAATTTGAGAGAGTGTGGCCAGGCAG	
**mCherry-Cald(1-200/374-531):**		pPL1678
Forward	CGAAAATCAGAAGTGCTTCACTCCTAAAGGCTCATCTCTC	
Reverse	AGCACTTCTGATTTTCGCCAAGGTTCCCTCCC	
**mCherry-Cald(1-200/427-531):**		pPL1679
Forward	CGAAAATCAGACAAAAGCTTCAAAACCTATGAAGCCTGC	
Reverse	GAAGCTTTTGTCTGATTTTCGCCAAGGTTCCCTCCC	
**mCherry-Cald(1-200/453-531):**		pPL1680
Forward	CGAAAATCAGATGTGGGAGAAAGGGAGTGTGTTTTCATC	
Reverse	TCTCCCACATCTGATTTTCGCCAAGGTTCCCTCCC	
**mCherry-Cald(1-200/498-531):**		pPL1681
Forward	AAAATCAGCCCGCTCCCAAGCCTTCTGAC	
Reverse	TGGGAGCGGGCTGATTTTCGCCAAGGTTCCCTCCC	
**mCherry-FL-H-Cald:**		pPL1944
Forward	GTCCGGACTCAGATCTCGAGTGACCACCATGGATGATTTTGAGCGTCGCAGAGAAC	

Reverse	TGATCAGTTATCTAGATCCGGTGGATCCTCAAACCTTAGTGGGGGAAGTGACCTTATC	
**Cald protein constructs:**		
**pHis10-SUMO-Cald(2-531):**		pPL1700
Forward	AGCAGCAGACGGGAGGGCTTAGCAGATCCGGGTCCCAGGG	
Reverse	CTTTGTTAGCAGCCGGATCTCAGACCTTAGTGGGAGAAGTGACC	
**pHis10-SUMO-Cald(197-531):**		pPL1702
Forward	AGCAGCAGACGGGAGGGGGCGAAAATCAGATCAAAGATGAGAAG	
Reverse	CTTTGTTAGCAGCCGGATCTCAGACCTTAGTGGGAGAAGTGACC	
**pHis10-SUMO-Cald(2-200)**		pPL1701
Forward	AGCAGCAGACGGGAGGGCTTAGCAGATCCGGGTCCCAGGG	
Reverse	CTTTGTTAGCAGCCGGATCTCACTGATTTTCGCCAAGGTTCCCTCCC	
**pHis10-SUMO-Cald(380-531):**		PL1705
Forward	AGCAGCAGACGGGAGGGGGCTCATCTCTCAAGATAGAGGAGCGAG	
Reverse	CTTTGTTAGCAGCCGGATCTCAGACCTTAGTGGGAGAAGTGACC	
