# Supplementary material for: Caldesmon controls stress fiber force-balance through dynamic cross-linking of myosin II and actin-tropomyosin filaments
Source: Nat Commun. 2022 Oct 13;13:6032. doi: 10.1038/s41467-022-33688-w (PMC9561149; doi:10.1038/s41467-022-33688-w)
Supplement: Supplementary file 3 — Description of additional Supplementary File [file 41467_2022_33688_MOESM3_ESM.pdf]

Descriptions of Additional Supplementary Files

Supplementary Movie 1: Wound healing assay example of wild-type U2OS cells. Representative wound healing assay time-lapse imaging example of wild-type U2OS cells seeded on fibronectincoated 12 well plate. Time-lapse images, after creation of a wound on a uniform monolayer of wildtype cells, were captured by ImageXpress® Nano Automated Imaging System (Molecular Devices) with 10 min interval for 24 h. Video is related to Figure 3g (upper panel) and Figure 3h (blue circles). The time-lapse images indicate complete wound closure in wild-type cells following 24 h of wound creation. Video play rate is 10 frames/ s. Scale bars, 400 µm.

Supplementary Movie 2: Wound healing assay example of Caldesmon KO1 U2OS cell. Representative wound healing assay time-lapse imaging example of Caldesmon KO1 U2OS cells seeded on fibronectin-coated 12 well plate. Time-lapse images, after creation of a wound on uniform monolayer of Caldesmon KO1 cells, were captured by ImageXpress® Nano Automated Imaging System (Molecular Devices) with 10 min interval for 24 h. Video is related to Figure 3g (lower panel) and Figure 3h (grey triangles). The time-lapse images indicate incomplete wound closure in Caldesmon KO1 cells following 24 h of wound creation as compared to wild-type cells. Video play rate is 10 frames/ s. Scale bars, 400 µm.

Supplementary Movie 3: Wound healing assay example of Caldesmon KO2 U2OS cell. Representative wound healing assay time-lapse imaging example of Caldesmon KO2 U2OS cells seeded on fibronectin-coated 12 well plate. Time-lapse images, after creation of a wound on uniform monolayer of Caldesmon KO2 cells, were captured by ImageXpress® Nano Automated Imaging System (Molecular Devices) with 10 min interval for 24 h. Video is related to Figure 3h (Orange squares). The time-lapse images indicate incomplete wound closure in Caldesmon KO2 cells following 24 h of wound creation as compared to wild-type cells. Video play rate is 10 frames/ s. Scale bars, 400 µm.

Supplementary Movie 4: Retrograde flow of transverse arcs in GFP-Lifeact-transfected wild-type U2OS cell. Live-cell imaging of a wild-type U2OS cell transfected with GFP-Lifeact plasmid 24 h before seeding on unpatterned fibronectin-coated dishes. Wild-type cell display uniform centripetal flow of transverse arcs, which later fuse to form thicker contractile bundles. Video is related to Supplementary Figure S5f. Time-lapse imaging was performed using 3I Marianas imaging system (3I intelligent Imaging Innovations) with 10 s interval for 30 min. Video play rate is 5 frames/ s. Scale bars, 10 µm.

Supplementary Movie 5: Retrograde flow of transverse arcs in GFP-Lifeact-transfected Caldesmon KO U2OS cell. Live-cell imaging of a Caldesmon KO U2OS cell transfected with GFP-lifeact plasmid 24 h before seeding on unpatterned fibronectin-coated dishes. Although Caldesmon KO cells showed centripetal flow of transverse arcs, the arcs displayed relatively slow flow parallel to each other. Video is related to Supplementary Figure S5g. Time-lapse imaging was performed using 3I Marianas imaging system (3I intelligent Imaging Innovations) with 10 s interval for 30 min. Video play rate is 5 frames/ sec. Scale bars, 10 µm.

Supplementary Movie 6: Retrograde flow of transverse arcs in GFP-Lifeact-transfected wild-type U2OS cell on a circular micropattern. Live-cell imaging of a wild-type U2OS cell transfected with GFP-Lifeact plasmid 24 h before seeding on fibronectin-coated dish with circular micropatterns. Video is related to Figure 4c (upper panel). Time-lapse imaging was performed using 3I Marianas imaging system (3I

intelligent Imaging Innovations) with 10 s interval for 20 min. Video play rate is 5 frames/ s. Scale bars, 10 µm.

Supplementary Movie 7: Retrograde flow of transverse arcs in GFP-Lifeact-transfected Caldesmon KO U2OS cell on a circular micropattern. Live-cell imaging of a Caldesmon KO U2OS cell transfected with GFP-lifeact plasmid 24 h before seeding on fibronectin-coated dish with circular micropatterns. Video is related to Figure 4c (lower panel). Time-lapse imaging was performed using 3I Marianas imaging system (3I intelligent Imaging Innovations) with 10 s interval for 20 min. Video play rate is 5 frames/ sec. Scale bars, 10 µm.

Supplementary Movie 8: NM-IIA distribution in wild-type U2OS cell. Representative zoomed-in time lapse images of a wild-type U2OS cell transfected with GFP-myosin II construct, and seeded on fibronectin-coated 35 mm glass-bottomed imaging dishes for 2 h before imaging using Zeiss LSM 880 upright Airyscan confocal microscope. The time-lapse images were captured for 30 min with 10 s interval. Wild-type cells display uniformly distributed myosin II filaments along the stress fibers, which display movement towards the cell center. Video related to Figure 6d (upper panel). Video play rate is 10 frames/ s. Scale bars, 5 µm.

Supplementary Movies 9 and 10: Two examples of NM-IIA distribution in Caldesmon KO U2OS cell. Representative zoomed-in time lapse imaging examples of Caldesmon KO U2OS cells transfected with GFP-myosin II construct, and seeded on fibronectin-coated 35 mm glass-bottomed imaging dishes for 2 h before imaging using Zeiss LSM 880 upright Airyscan confocal microscope. The time-lapse images were captured for 30 min with 10 s interval. Caldesmon KO cells display abnormal lateral sliding of myosin II filaments, and consequent accumulation of myosin at various regions along contractile stress fibers. Video related to Figure 6d (lower panel). Video play rate is 10 frames/ s. Scale bars, 5 µm.
