# Supplementary material for: Caldesmon controls stress fiber force-balance through dynamic cross-linking of myosin II and actin-tropomyosin filaments
Source: Nat Commun. 2022 Oct 13;13:6032. doi: 10.1038/s41467-022-33688-w (PMC9561149; doi:10.1038/s41467-022-33688-w)
Supplement: Supplementary file 14 — Reporting Summary [file 41467_2022_33688_MOESM14_ESM.pdf]

nature portfolio

Corresponding author(s): Pekka Lappalainen

Last updated by author(s): Sep 15, 2022

Reporting Summary

Nature Portfolio wishes to improve the reproducibility of the work that we publish. This form provides structure for consistency and transparency in reporting. For further information on Nature Portfolio policies, see our Editorial Policies and the Editorial Policy Checklist.

Statistics

For all statistical analyses, confirm that the following items are present in the figure legend, table legend, main text, or Methods section.

n/a	Confirmed	
☐	☒	The exact sample size (*n*) for each experimental group/condition, given as a discrete number and unit of measurement
☐	☒	A statement on whether measurements were taken from distinct samples or whether the same sample was measured repeatedly
☐	☒	The statistical test(s) used AND whether they are one- or two-sided<br>*Only common tests should be described solely by name; describe more complex techniques in the Methods section.*
☐	☒	A description of all covariates tested
☐	☒	A description of any assumptions or corrections, such as tests of normality and adjustment for multiple comparisons
☐	☒	A full description of the statistical parameters including central tendency (e.g. means) or other basic estimates (e.g. regression coefficient) AND variation (e.g. standard deviation) or associated estimates of uncertainty (e.g. confidence intervals)
☐	☒	For null hypothesis testing, the test statistic (e.g. *F*, *t*, *r*) with confidence intervals, effect sizes, degrees of freedom and *P* value noted<br>*Give P values as exact values whenever suitable.*
☒	☐	For Bayesian analysis, information on the choice of priors and Markov chain Monte Carlo settings
☒	☐	For hierarchical and complex designs, identification of the appropriate level for tests and full reporting of outcomes
☒	☐	Estimates of effect sizes (e.g. Cohen's *d*, Pearson's *r*), indicating how they were calculated

Our web collection on statistics for biologists contains articles on many of the points above.

Software and code

Policy information about availability of computer code

Data collection	Caldesmon knockout cells were sorted individually with FACSAria II cell sorter (BD) supplied with BD FACSDiVa Software v 8.0 software. Western blot images were acquired using ChemiDoc XRS+ System (1708265, Biorad) supplied with Image Lab™ 6.0 (Biorad) software. Wide-field, confocal and super resolution imaging data were collected using Leica Application Suite X (3.3.0.16799 version), Fusion 2.0 iXon SRRF-Stream real time super-resolution and AquireSR 4.4 software, respectively. Super-resolution images were reconstructed and aligned using SoftWoRx 7.0 software. For live cell imaging of myosin dynamics and retrograde flow, as well as for laser nanosurgery experiments, Zeiss Zen 2.0 and ZEN black software were used. Fluorescence recovery after photobleaching (FRAP) experiments, measurement of retrograde flow of transverse arcs, and traction force microscopy (TFM) were performed using Zeiss Zen 2.0 and Slidebook 6.0.15 software. Random migration assay and wound-healing assay were performed using Cell-IQ Imagen 4.1.0 software.

Data analysis	For densitometric quantifications of Western blots and co-sedimentation assay gels, Image Lab™ 6.0 (Biorad) and Fiji imageJ 1.53c were used. For the analysis of cell shape and size, high-content image analysis was done using CellProfiler 3.1.8 automated image analysis software. FRAP image analysis was performed using Slidebook 6.0.15 software. The analysis of random migration and wound-healing assay images were done using Cell-IQ Analyser 4.4.0 software. Single blind analysis of TFM, retrograde arc flow, and laser ablation studies were performed using Fiji ImageJ 1.53c. The kymograph analysis of transverse arcs was done using Fiji ImageJ 1.53c. Blind image analysis of myosin distribution was performed using windows image Fiji ImageJ 1.53c and windows image viewer. The quantification of YAP-localization in cytoplasm vs. nucleus was performed using Fiji ImageJ-ROI tool. DiAna co-localization analyses were performed using Fiji imageJ 1.53c-based 3D-iterative segmentation, 3D-imageJ suite and DiAna tools. All the values obtained after quantifications were recorded, analyzed and calculated in spreadsheets using Microsoft excel version-2016. All the graphical representations were designed using GraphPad Prism 7.03 software. GraphPad Prism 7.03 software was further used for determining the statistical significance by one-way/ two-way ANOVA in Tukey's post hoc HSD analysis, multiple comparison tests and Mann-Whitney/ Wilcoxon rank-sum test (MWW).

For manuscripts utilizing custom algorithms or software that are central to the research but not yet described in published literature, software must be made available to editors and reviewers. We strongly encourage code deposition in a community repository (e.g. GitHub). See the Nature Portfolio guidelines for submitting code & software for further information.

Data

Policy information about availability of data

All manuscripts must include a data availability statement. This statement should provide the following information, where applicable:

- Accession codes, unique identifiers, or web links for publicly available datasets
- A description of any restrictions on data availability
- For clinical datasets or third party data, please ensure that the statement adheres to our policy

The data supporting the findings of the study are available in the manuscript and supplementary information. Other raw data generated in the study are provided as source files and would be available from the corresponding author on reasonable request.

Human research participants

Policy information about studies involving human research participants and Sex and Gender in Research.

Reporting on sex and gender	N/A
Population characteristics	N/A
Recruitment	N/A
Ethics oversight	N/A

Note that full information on the approval of the study protocol must also be provided in the manuscript.

Field-specific reporting

Please select the one below that is the best fit for your research. If you are not sure, read the appropriate sections before making your selection.

- [x] Life sciences
- [ ] Behavioural & social sciences
- [ ] Ecological, evolutionary & environmental sciences

For a reference copy of the document with all sections, see nature.com/documents/nr-reporting-summary-flat.pdf

Life sciences study design

All studies must disclose on these points even when the disclosure is negative.

Sample size	All experiments were performed at least in triplicates, unless mentioned in the manuscript text, with the indicated sample sizes in each individual repeat. No sample size calculations were performed for the study. The sample size were decided arbitrarily and were considered sufficient as the overall phenotypes within groups could be identified comparatively.
Data exclusions	The cells with very high expression of GFP-actin, and thus displaying abnormal actin cytoskeleton arrangement, were excluded from the FRAP and transverse arc flow experiments. For laser nanosurgery experiments, retraction curves were excluded from the tau and L0 datasets if they did not meet all of the following criteria for the fitted KV equation: 1) adjusted R2 > 0.9, 2) tau < 0.8 * imaging window (tau < 60), and 3) sum squared errors of prediction < 10. In TFM experiments, those cells that were not properly attached to the substrate were excluded from the analysis. In wound healing assays, subconfluent areas were excluded from imaging, and in random cell migration assays the cells that attached to each other were excluded from the analysis.
Replication	All the experimental replicates were performed independently as per the protocols established for the respective experiments. The observations from the first replicate were recorded and were considered during the analysis of the further repeats. Similar observations were seen within independent repeats of every experiment.

Randomization	Imaging experiments for determining NM-IIA filament phenotypes in wild-type U2OS and Caldesmon knockout cells were performed in a randomized manner by mixing wild-type and knockout cells with each other. The cells were stained with phalloidin, as well as with NMII and Caldesmon -specific antibodies to compare the NMII phenotypes of wild-type vs. Caldesmon knockout cells. Similar procedure was also used in YAP localization experiments (with YAP and Caldesmon -specific antibodies).
Blinding	For YAP localization experiments, the localization of YAP was analyzed blindly without knowing which cells are wild-type and Caldesmon knockout cells. Blind analysis was performed for determining the NM-IIA filament phenotype in wild-type and Caldesmon knockout U2OS cells and for the analysis of NM-IIA filament rescue phenotype in Caldesmon FL-constructs of L-and H-Caldesmon and domain-deletion construct expressing knockout cells.

Reporting for specific materials, systems and methods

We require information from authors about some types of materials, experimental systems and methods used in many studies. Here, indicate whether each material, system or method listed is relevant to your study. If you are not sure if a list item applies to your research, read the appropriate section before selecting a response.

Materials & experimental systems

n/a	Involved in the study
☐	☒ Antibodies
☐	☒ Eukaryotic cell lines
☒	☐ Palaeontology and archaeology
☒	☐ Animals and other organisms
☒	☐ Clinical data
☐	☒ Dual use research of concern

Methods

n/a	Involved in the study
☒	☐ ChIP-seq
☐	☒ Flow cytometry
☒	☐ MRI-based neuroimaging

Antibodies

Antibodies used	Anti-Caldesmon/CDM antibody, company- Abcam, catalogue number-ab32330, clone name- E89, lot number- GR1376472, anti-L-Caldesmon, company- Santa Cruz Biotechnology, catalogue no- sc-48427, clone name- F-1, lot number- #J1906, anti-Vinculin, company- Sigma-Aldrich, catalogue number- V9131, clone name- hVIN-1, lot number- 079M4754V, anti-NM-IIA, company- Biolegend, catalogue number- 909801, lot number- B292969, anti-RLC, company- Sigma-Aldrich, catalogue number- M4401, clone name- MY-21, lot number- 046M818V, anti-phospho-MLC (Thr18, Ser19), company- Cell Signaling Technology, catalogue number- 3671, lot number- 6, anti-alpha actinin-1, company- Sigma-Aldrich, catalogue number- A5044, anti-YAP, company- Novus, catalogue number- NB10-58358SS, lot number- B-5, anti-GAPDH, company- Sigma-Aldrich, catalogue number- G9545, lot number- #099M4801V, anti-Tropomyosin 1 and 2, company- Sigma-Aldrich, catalogue number- T2780, clone name- TM311, lot number- #014M4782. Anti-Tropomyosin 2 and 4 (LC24) and anti-tropomyosin 3 (γ-9d) antibodies were kind gifts from Peter W. Gunning (University of New South Wales, Sydney, Austarlia). Goat anti-rabbit HRP-conjugate, company- Thermo Fisher Scientific, catalogue number- G21234, Goat anti-mouse HRP-conjugate, company- Thermo Fisher Scientific, catalogue number- 31430, Alexa Fluor goat anti-mouse 488, company- Thermo Fisher Scientific, catalogue number- A11001, lot number- 2015565, Alexa Fluor goat anti-mouse 568, company- Thermo Fisher Scientific, catalogue number- A11031, lot number- 2124366, Alexa Fluor goat anti-mouse 647, company- Thermo Fisher Scientific, catalogue number- A11001, lot number- 1839633, Alexa Fluor goat anti-rabbit 488, company- Thermo Fisher Scientific, catalogue number- A11034, lot number- 1937195, Alexa Fluor goat anti-rabbit 568, company- Thermo Fisher Scientific, catalogue number- A11011, lot number- 1811756, Alexa Fluor goat anti-rabbit 647, company- Thermo Fisher Scientific, catalogue number- A32733, lot number- UC279417. Alexa Fluor phalloidin 488 (1:400; A12379), phalloidin 568 (1:400; A1238) and phalloidin 647 (1:400; A22287).
Validation	All the antibodies were used as per the manufacturer's recommendations.

Eukaryotic cell lines

Policy information about cell lines and Sex and Gender in Research

Cell line source(s)	Human osteosarcoma (U2OS) cells were used in this study. The cell-line was from ATCC (Cat# HTB-96, RRID:CVCL_0042).
Authentication	The cell-line has been authenticated through STR profiling.
Mycoplasma contamination	The U2OS wild-type cells and Caldesmon knockout clones were regularly tested for mycoplasma contamination using Lonza™ Mycoalert™ Mycoplasma Detection Kit, company- Thermo Fisher Scientific, catalogue number- LT07-418. The cell lines were tested negative for mycoplasma contamination.
Commonly misidentified lines (See ICLAC register)	This study did not use any misidentified cell lines.

Dual use research of concern

Policy information about dual use research of concern

Hazards

Could the accidental, deliberate or reckless misuse of agents or technologies generated in the work, or the application of information presented in the manuscript, pose a threat to:

No	Yes	
☒	☐	Public health
☒	☐	National security
☒	☐	Crops and/or livestock
☒	☐	Ecosystems
☒	☐	Any other significant area

Experiments of concern

Does the work involve any of these experiments of concern:

No	Yes	
☒	☐	Demonstrate how to render a vaccine ineffective
☒	☐	Confer resistance to therapeutically useful antibiotics or antiviral agents
☒	☐	Enhance the virulence of a pathogen or render a nonpathogen virulent
☒	☐	Increase transmissibility of a pathogen
☒	☐	Alter the host range of a pathogen
☒	☐	Enable evasion of diagnostic/detection modalities
☒	☐	Enable the weaponization of a biological agent or toxin
☒	☐	Any other potentially harmful combination of experiments and agents

Flow Cytometry

Plots

Confirm that:

☒ The axis labels state the marker and fluorochrome used (e.g. CD4-FITC).

☒ The axis scales are clearly visible. Include numbers along axes only for bottom left plot of group (a 'group' is an analysis of identical markers).

☒ All plots are contour plots with outliers or pseudocolor plots.

☒ A numerical value for number of cells or percentage (with statistics) is provided.

Methodology

Sample preparation	The Caldesmon knockout clones were treated with 0.05% trypsin-EDTA solution for 5 min at 37 degrees and 5% CO2 conc. Then trypsinized cells were suspended in DMEM complete media and pelleted at 750Xg for 4 min at room temperature. The cell pellets were re-suspended in appropriate amount of DMEM complete media containing 25 mM HEPES pH-7.5 such that the cell concentration is between 5 - 7.5 x 106 cells/ml. Finally, the cells were passed through a cell strainers to collect into 5 ml polystyrene FACS tubes before performing FACS acquisition.
Instrument	BD FACSAria IIIu.
Software	BD FACSDiVa Software v 8.0.
Cell population abundance	The CRISPR/Cas9 expression plasmid-pSpCas9(BB) containing oligo1 and oligo2 guides for the Caldesmon deletion expressed the GFP, which was used as a selection marker for sorting only the transfected cells. Caldesmon oligo1 and oligo2 KO cells were 3.7% and 3.3% of the overall cell population.

Gating strategy

For sorting, 75-100 um nozzle size was selected. Out of the total cell population, the initial settings for FSC/SSC adjustments were performed using 5000 events such that only moderately-expressing healthy cell population was further selected separating out cell debris. Non-transfected U2OS cells were sorted first to mark gates to exclude negative population or cells showing deam expression of KO-plasmids. Based on the settings GFP-positive Caldesmon knockout cells were loaded for sorting. The population of interest was marked in the gate P1 against FSC-A/SSC-A scatter plot. Gate 2, 3 and 4 were marked from the selected population further to exclude deam/ high-GFP expressing cells. Finally oligo1 and oligo2 knockout cells were sorted into single 96 well plates simultaneously.

☒ Tick this box to confirm that a figure exemplifying the gating strategy is provided in the Supplementary Information.
