# Supplementary material for: Caldesmon controls stress fiber force-balance through dynamic cross-linking of myosin II and actin-tropomyosin filaments
Source: Nat Commun. 2022 Oct 13;13:6032. doi: 10.1038/s41467-022-33688-w (PMC9561149; doi:10.1038/s41467-022-33688-w)
Supplement: Supplementary file 15 — Source Data [file 41467_2022_33688_MOESM15_ESM.zip › SourceDataFile_Gels.pdf]

Source data: Supplementary Figure 7b

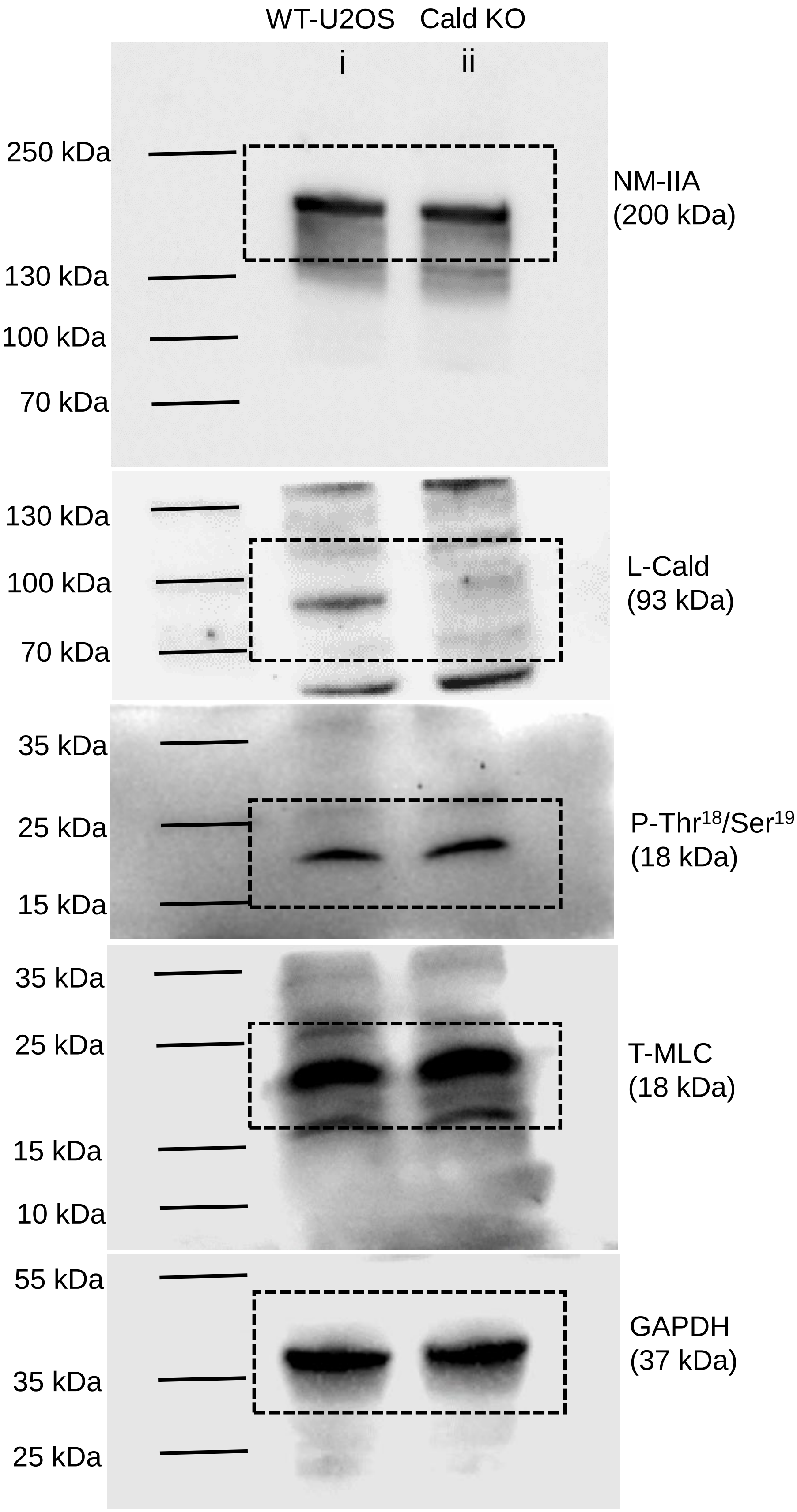

Supplementary unprocessed Western blots: Anti-NMIIA, anti-L-Cald, anti-P-Thr^{18}/Ser^{19}, anti-T-MLC and anti-GAPDH blots of Supplementary Figure 7b. Please note that some membranes were cut before chemiluminiscence detection. Protein bands were determined based on antibody datasheets from the manufacturer, and by comparing the detected antibody bands the with protein markers determined by epi-white illumination.

Source data: Supplementary Figure 7h

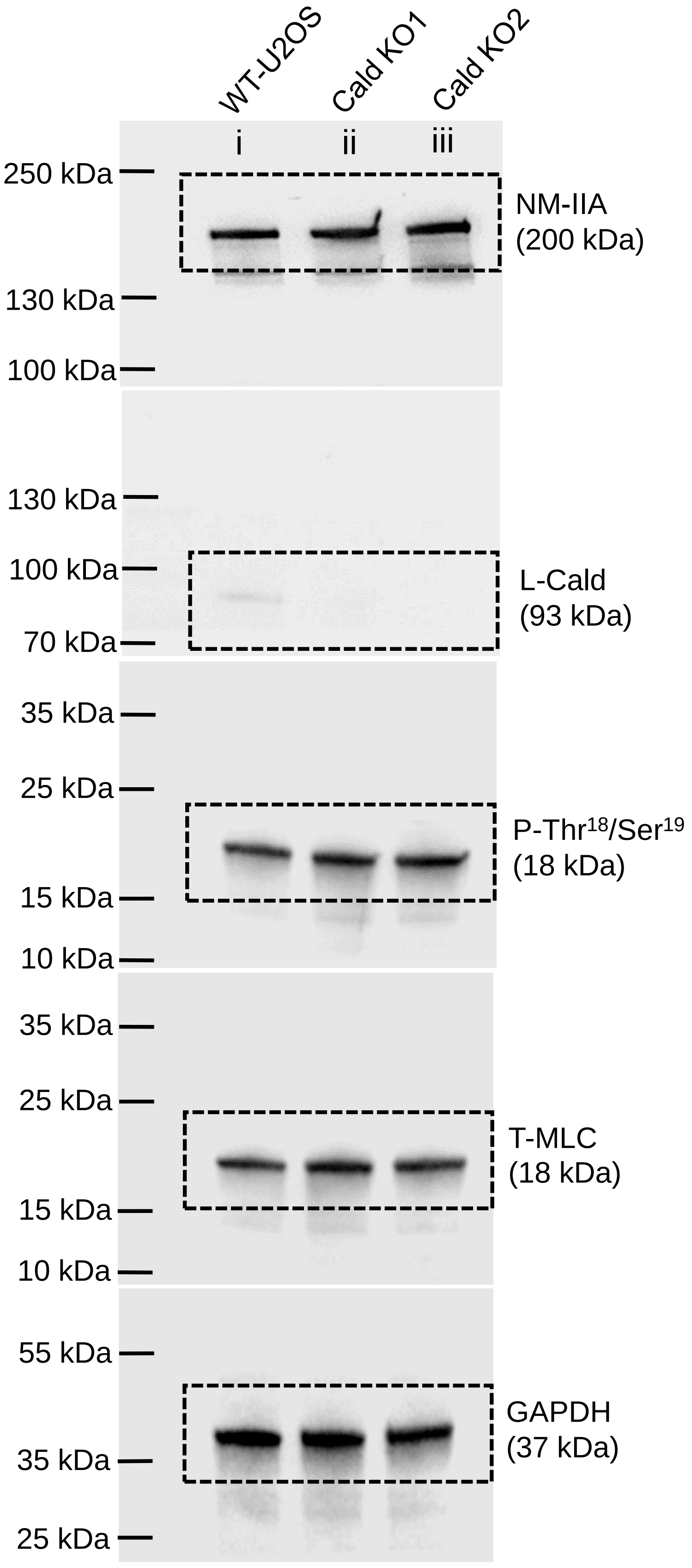

Supplementary unprocessed Western blots: Anti-NMIIA, anti-L-Cald, anti-P-Thr^{18}/Ser^{19}, anti-T-MLC and anti-GAPDH blots of Supplementary Figure 7h. Please note that the membranes were cut before chemiluminiscence detection and protein bands were determined based on antibody datasheets from the manufacturer, and by comparing detected antibody bands with protein markers determined by epi-white illumination.

Source data: Figure 3a

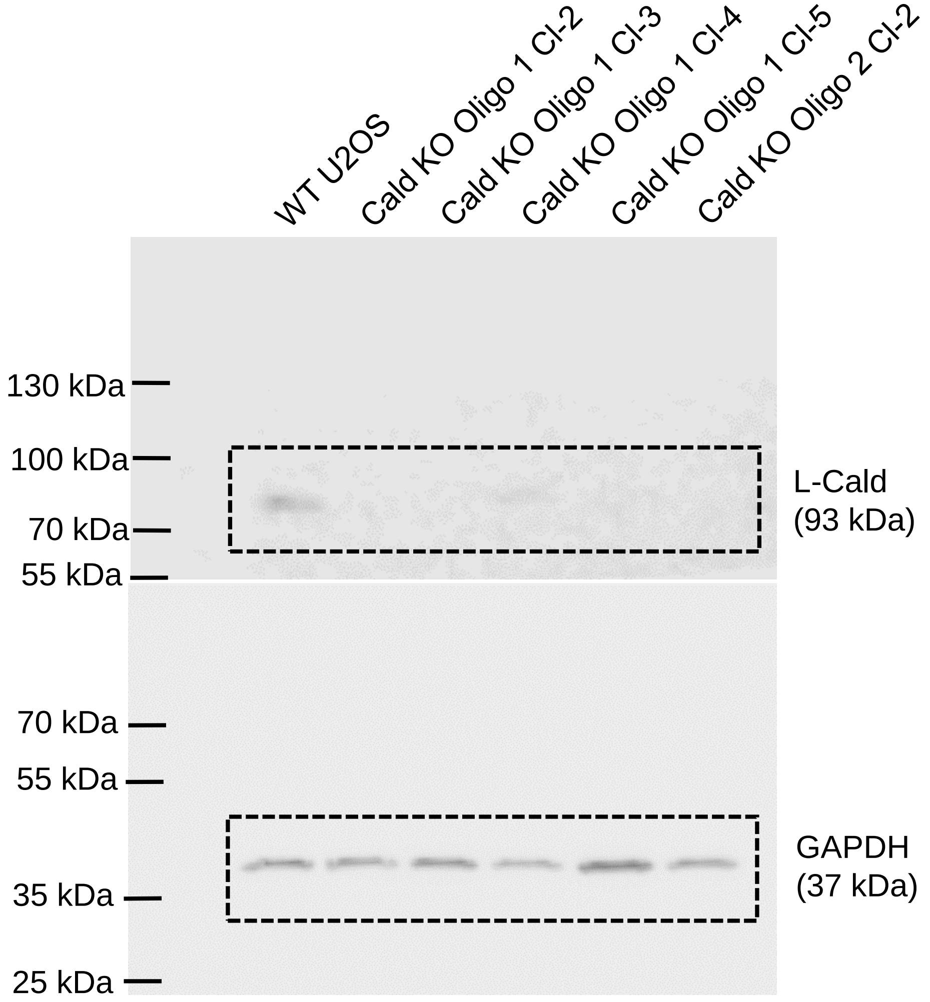

Supplementary unprocessed Western blots: Anti-L-Cald,and anti-GAPDH blots of Figure 3a. Please note that the membranes were cut before the Chemiluminiscence detection and protein bands were determined based on antibody datasheets from the manufacturer, and by comparing detected antibody bands with protein markers determined by epi-white illumination.
